# Molecular Mechanisms of Curcumin Renoprotection in Experimental Acute Renal Injury

**DOI:** 10.3389/fphar.2017.00912

**Published:** 2017-12-12

**Authors:** Youling Fan, Hongtao Chen, Huihua Peng, Fang Huang, Jiying Zhong, Jun Zhou

**Affiliations:** ^1^Department of Anesthesiology, Panyu Central Hospital, Guangzhou, China; ^2^Department of Anesthesiology, The Eighth People's Hospital of Guangzhou, Guangzhou, China; ^3^Department of Anesthesiology, The First People's Hospital of Foshan, Foshan, China

**Keywords:** acute kidney injury, curcumin, APPL1, apoptosis, Akt

## Abstract

As a highly perfused organ, the kidney is especially sensitive to ischemia and reperfusion. Ischemia-reperfusion (IR)-induced acute kidney injury (AKI) has a high incidence during the perioperative period in the clinic and is an important link in ischemic acute renal failure (IARF). Therefore, IR-induced AKI has important clinical significance and it is necessary to explore to develop drugs to prevent and alleviate IR-induced AKI. Curcumin [diferuloylmethane, 1,7-bis(4-hydroxy-3-methoxiphenyl)-1,6-heptadiene-3,5-dione)] is a polyphenol compound derived from *Curcuma longa* (turmeric) and was shown to have a renoprotective effect on ischemia-reperfusion injury (IRI) in a previous study. However, the specific mechanisms underlying the protective role of curcumin in IR-induced AKI are not completely understood. APPL1 is a protein coding gene that has been shown to be involved in the crosstalk between the adiponectin-signaling and insulin-signaling pathways. In the study, to investigate the molecular mechanisms of curcumin effects in kidney ischemia/reperfusion model, we observed the effect of curcumin in experimental models of IR-induced AKI and we found that curcumin treatment significantly increased the expression of APPL1 and inhibited the activation of Akt after IR treatment in the kidney. Our *in vitro* results showed that apoptosis of renal tubular epithelial cells was exacerbated with hypoxia-reoxygenation (HR) treatment compared to sham control cells. Curcumin significantly decreased the rate of apoptosis in renal tubular epithelial cells with HR treatment. Moreover, knockdown of APPL1 activated Akt and subsequently aggravated apoptosis in HR-treated renal tubular epithelial cells. Conversely, inhibition of Akt directly reversed the effects of APPL1 knockdown. In summary, our study demonstrated that curcumin mediated upregulation of APPL1 protects against ischemia reperfusion induced AKI by inhibiting Akt phosphorylation.

## Introduction

Ischemia reperfusion (IR) is the leading cause of acute kidney injury (AKI), which is one of the most serious and common health problems in the clinic (Malek and Nematbakhsh, 2015). Currently, the prevention and treatment modalities of IR-induced AKI are neither ideal nor optimistic, as the specific molecular mechanism of IR-induced AKI remains elusive. Ample evidence has suggested that tubular necrosis/apoptosis is an important mechanism underlying ischemia-induced AKI (Saikumar and Venkatachalam, 2003; Toronyi, 2008). In the pathogenesis of IR-induced AKI, inflammatory and immune cell infiltration as well as altered chemokine and cytokine production lead to the apoptosis and necrosis of renal tubular epithelial cells (Ornellas et al., 2017). Therefore, a better understanding of the cellular and molecular mechanisms of apoptosis underlying IR-induced AKI is needed. Furthermore, medications or treatment strategies that reduce apoptosis-related kidney damage are essential for developing effective therapies.

Curcumin is a type of fat soluble phenolic pigment extracted from the turmeric rhizome. The molecular formula is C_21_H_20_O_6_ and the absolute molecular mass is 368.37 (Esatbeyoglu et al., 2012). The biological effects of curcumin range from anti-inflammatory, antioxidant, anti-fibrosis, anticoagulation, and specific antitumor activity, and it has also been found to possess specific organ protective effects (Park et al., 2016; Kunnumakkara et al., 2017). In recent years, an increasing number of scholars have studied the renoprotective effects of curcumin. The molecular mechanism underlying its varied cellular effects has been studied in some detail. Curcumin has been shown to down-regulate the NFkB signaling pathway thereby inducing apoptosis, which has been found be an important mechanism of AKI (Ozkok et al., 2016). However, the specific renoprotective effect and the cellular and molecular mechanism of curcumin in the pathogenesis of IR-induced AKI remain unclear.

An adaptor protein, phosphotyrosine interacting with PH domain and leucine zipper 1 (APPL1), is a cohesion protein for adiponectin receptors (adiponectin receptor, AdipoR) that can directly interact with the intracellular N end of AdipoR and participate in adiponectin cell signaling (Diggins and Webb, 2017). Increasing evidence has indicated that the APN signaling pathway is involved in AKI and recent evidence directly suggested that APPL1 has a protective role in multiple organs with acute injury (Ji et al., 2015; XiaoTian et al., 2016). Therefore, we speculated that APPL1 may play an important role in IR-induced AKI. In the present study, to investigate the molecular mechanisms of curcumin effects in kidney ischemia/reperfusion model, we observed the effect of curcumin in experimental models of IR-induced AKI and we found that curcumin has a nephroprotective role that is accompanied by upregulation of APPL1 expression and inhibition of Akt activity in the kidney in response to IRI.

Therefore, we investigated whether curcumin mediates APPL1 activity to inhibit Akt phosphorylation and alleviate apoptosis in renal tubular epithelial cells treated with hypoxia-reoxygenation (HR) *in vitro*. Our results demonstrate that curcumin mediates the upregulation of APPL1 to reduce apoptosis and protect against ischemia reperfusion induced AKI through inhibition of Akt phosphorylation.

## Methods

### Animals

The animal experiments were conducted according to the Guide for the Care and Use of Laboratory Animals and were approved by the Institutional Animal Care and Use Committee of the First People Hospital of Foshan. Male BALB/c mice at 8–12 weeks of age, weighing 20–30 g, were anesthetized by intraperitoneal injection of ketamine (80 mg/kg) and xylazine (10 mg/kg). Kidneys were exposed through flank incision and subjected to ischemia by clamping of the renal pedicles with non-traumatic microaneurism clamps. The time of ischemia was chosen to obtain a reversible model of ischemic acute renal failure (IARF) and to avoid animal mortality. After 30 min, the clamps were removed and blood reflow was confirmed. Body temperature was maintained at 36.5–37.5°C throughout the procedure. Mice were randomly allocated into the following groups: (1) NC+sham group (*n* = 8); (2) NC+I/R group (*n* = 8); (3) Curcumin+sham group (*n* = 8); and (4) Curcumin+I/R group (*n* = 8). Sham control mice underwent an identical surgical procedure but without pedicle clamping. Mice were administered curcumin (100 mg/kg) 0.5 h before I/R induction. All animals were sacrificed at 24 h after reperfusion. Kidneys were perfused and harvested.

### Measurement of renal function

Blood samples were obtained from the inferior vena cava 24 h after reperfusion. Serum creatinine was measured using a creatinine assay kit (BioAssay Systems, Hayward, CA) according to the manufacturer's instructions. Blood urea nitrogen was determined fluorometrically as described (Ramesh et al., 2007).

### Renal morphology

Kidney tissue was fixed in 10% buffered formalin, embedded in paraffin and then cut at 4 μm thickness. After deparaffinization and rehydration, sections were stained with H&E. Tissue damage was examined in a blinded manner and scored according to the percentage of damaged tubules as previously reported (Chen et al., 2011): 0, no damage; 1, <25% damage; 2, 25%−50% damage; 3, 50%−75% damage; and 4, >75% damage.

### Cell culture and experimental grouping

Mouse renal tubular epithelial cells (EpiCM-a) (Sciencell, San Diego, California, US) were thawed in a 38°C water bath and then centrifuged at 1,000 rpm for 5 min. The supernatant was discarded and the cells were cultured in DMEM medium in an incubator containing 5% CO_2_ at 37°C. The medium was replaced when cells adhered to the bottle wall. The cells were subcultured until 80% of the bottle bottom was covered with cells. To induce hypoxia, mouse renal tubular epithelial cells were incubated in serum-free DMEM in a hypoxia chamber containing 95% N2 and 5% CO_2_ at 37°C. Following exposure to hypoxic conditions, cell medium was replaced with fresh oxygenated DMEM and cells were reoxygenated for 24 h in normoxic conditions (5% CO^2^, 21% O_2_ and 74% N_2_) at 37°C.The cells with traces of APPL1 siRNA treatment were divided into six groups: Sham group, HR group, HR+Curcumin (25 μM) group, HR+Curcumin+APPL1 siRNA group, HR+Curcumin+NC siRNA group, and HR+Curcumin+APPL1 siRNA+AZD5363 (30 μM) group. HR treatment consisted of hypoxia for 3 h and then reoxygenation for 24 h. Curcumin and AZD5363 were administered at the beginning of HR treatment.

### APPL1 siRNA transfection

APPL1 siRNA was manufactured by Sigma (US). For each well that was transfected, RNAi duplex-Lipofectamine™ RNAiMAX complexes were prepared according to the following protocol: 6 pmol RNAi duplex in 100 μl Opti-MEM® I Medium was diluted without serum in the well of the tissue culture plate. Lipofectamine™ RNAiMAX was mixed gently before use, and then 1 μl Lipofectamine™ RNAiMAX was added to each well containing the diluted RNAi molecules. Cells were mixed gently and incubated for 10–20 min at room temperature. Cells were diluted in complete growth medium without antibiotics 24 h after plating. For suspension cells, 20,000–50,000 cells/well were used. In each well, 500 μl diluted cells were added with RNAi duplex—Lipofectamine™ RNAiMAX complexes. The final volume was 600 μl and the final RNA concentration was 10 nM. Cells were incubated for 24–72 h at 37°C in a CO_2_ incubator.

### NC siRNA transfection

NC siRNA is transfected with non-characteristic negative siRNA cells by transfection reagent.

### Immunohistochemistry

Immunohistochemical staining was performed on paraffin sections. Antigen retrieval was performed with antigen unmasking solution (Vector Laboratories) or proteinase K. Endogenous peroxidase activity was quenched with 3% H_2_O_2_ for 10 min. After blocking with 5% normal serum, slides were incubated with primary antibodies in a humidified chamber overnight. After washing, slides were incubated with appropriate secondary antibodies and ABC solution sequentially according to the ABC kit (Vector Laboratories). Slides were then visualized by incubation in diaminobenzidine solution for an appropriate duration of time. Nuclear staining was performed with hematoxylin. The slides were dehydrated, cleared, and mounted. The images from these slides were obtained and analyzed by NIS Element software (Nikon Instruments) with the Nikon microscope image system (Nikon Instruments).

### Detection of apoptotic cells in the kidneys

Apoptotic cell death was determined with terminal deoxynucleotidyl transferase–mediated dUTP nick-end labeling (TUNEL) staining with the DeadEnd Colorimetric Apoptosis Detection System (Millipore, Billerica, MA) according to the manufacturer's instructions. The number of TUNEL-positive cells per high-power field was counted and analyzed in a blinded fashion.

### Tunel assay with cells

Cells were added to 96-well plates (100 μL/well; 2 × 10^4^ cells/well) and incubated overnight, followed by cell transfection. After cells were treated with HR or sham, the medium was discarded and cells were fixed with 4% paraformaldehyde. Cells were incubated at room temperature for 30 min and washed with PBS. Permeabilization solution (0.1% Triton X-100 dissolved in 0.1% sodium citrate solution) was added to plates for a 2-min incubation in an ice-bath. With the addition of 50 μL TUNEL reagent, cells were cultured in a humidified incubator at 37°C for 60 min. After three PBS washes, 50 μL of DAPI was added to the cells, followed by incubation at 37°C in the dark and another three PBS washes. Cells were observed under a fluorescence microscope and images were captured.

### Flow cytometry analysis

An Annexin V-PE(V-phycoerythrin) and 7-AAD (7-amino-actinomycin D) double-staining Apoptosis Detection kit (KeyGEN, Nanjing, China) was used to detect apoptotic activity according to the manufacturer's instructions. Cells were harvested, washed, and incubated for 15 min with annexin V-FITC and PI. The cells were washed and transferred into 12 × 75-mm Falcon 2052 FACS tubes (Becton Dickinson, San Jose, CA), and data from cells were collected on FACS with a Becton Dickinson Biosciences FACScan and CellQuest software version 3.3. This combination allows for the differentiation between early apoptotic cells (annexin V-FITC positive, PI negative), late apoptotic and/or necrotic cells (annexin V and PI positive), and viable cells (unstained).

### Western blot analysis

Cells were washed with ice-cold PBS and lysed in ice-cold modified RIPA lysis buffer (50 mM Tris HCl, pH 7.5, 150 mM NaCl, 50 mM NaF, 0.5% deoxycholic acid, 1% NP-40, 1 mM sodium orthovana-date, 0.1% SDS). Insoluble material was removed by centrifugation at 12,000 × g for 15 min at 4°C. The protein concentrations were measured using a bicinchoninic acid assay (Perbio Science, Cramlington, UK) following the manufacturer's instructions. Protein was extracted using RIPA buffer containing a cocktail of proteinase inhibitors and quantified with a Bio-Rad protein assay. An equal amount of protein was separated on SDS-polyacrylamide gels in a Tris/SDS buffer system and then transferred onto nitrocellulose membranes. Blotting was performed according to standard procedures with primary antibodies overnight, followed by incubation with appropriate fluorescence-conjugated secondary antibodies. The proteins of interest were analyzed using an Odyssey IR scanner (LI-COR Biosciences). Signal intensities were quantified using NIH Image/J software (National Institutes of Health).

### Statistical analysis

The data were presented as the means ± SEM. Comparisons of multiple groups were performed by ANOVA followed by the Bonferroni *post-hoc* test. A *P* < 0.05 was considered statistically significant.

## Results

### Curcumin mitigates IR-induced AKI

**Figure 1:**
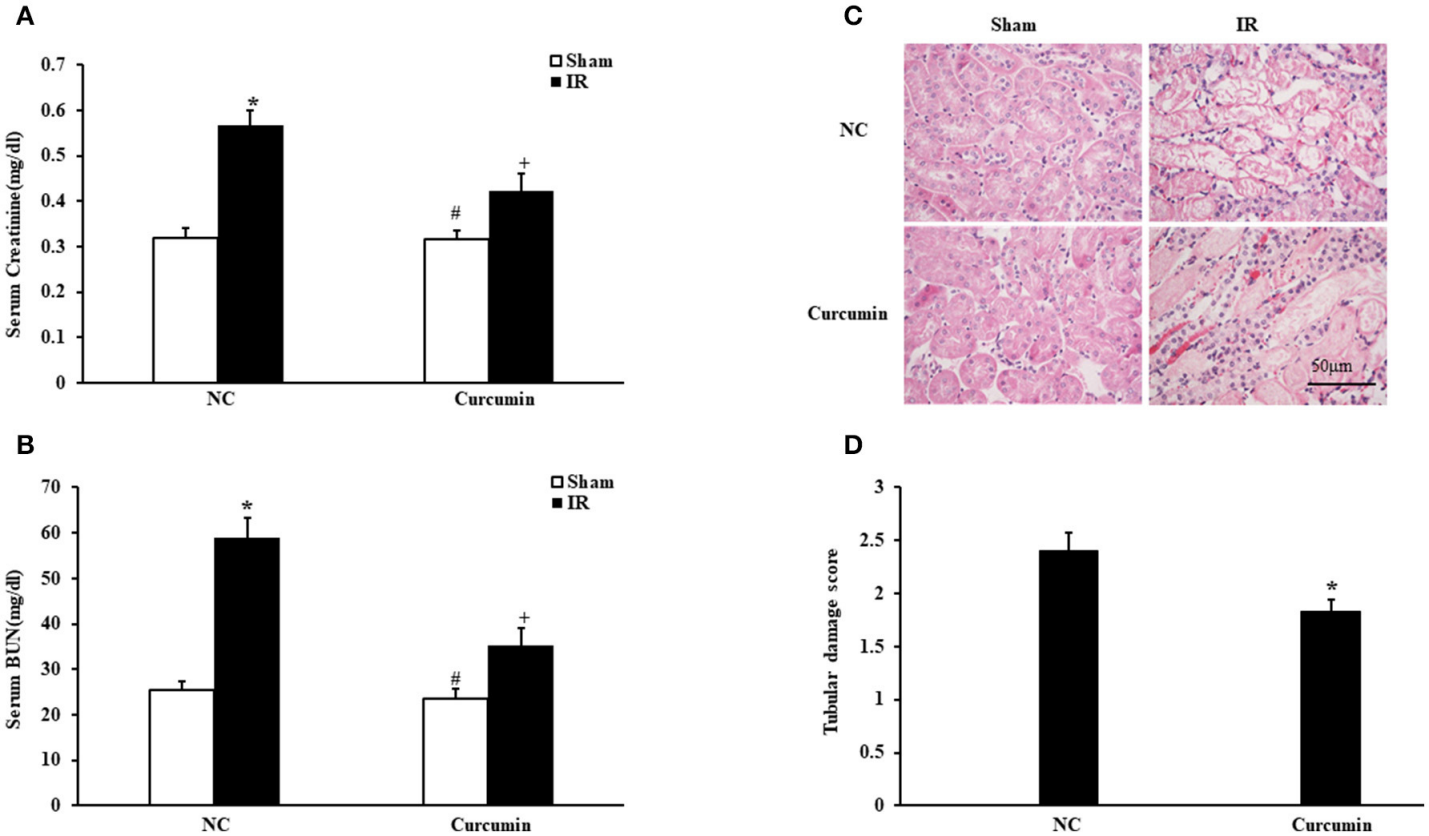
Curcumin mitigates IR-induced AKI. **(A)** Serum creatinine in NC and Curcumin group mice at 24 h after IR treatment. ^*^P < 0.05 vs. Sham treatment in the NC group; ^#^*P* < 0.05 vs. IR treatment in the Curcumin group; ^+^*P* < 0.05 vs. IR treatment in the NC group. *n* = 6 in each group. **(B)** Serum urea nitrogen in NC and Curcumin group mice at 24 h after IR treatment. ^*^P < 0.05 vs. Sham treatment in the NC group; ^#^*P* < 0.05 vs. IR treatment in the Curcumin group; ^+^*P* < 0.05 vs. IR treatment in the NC group. *n* = 6 in each group. **(C)** PAS staining for kidney sections of NC and Curcumin group mice at 24 h after IR or sham treatment. Scale bar: 50 μm. **(D)** Quantitative assessment of tubular damage in NC and Curcumin group mice at 24 h after IR treatment. ^*^P < 0.05 vs. IR treatment in NC group. *n* = 6 in each group.

We first determined if curcumin has nephroprotective action in a mouse model of kidney ischemia-reperfusion injury (IRI). BALB/c mice were treated i.p. with curcumin or equivalent normal saline (NS). Then, we detected the damage in the kidneys by assessing serum creatinine, urea nitrogen and histological injury. Renal dysfunction was induced as evidenced by a marked elevation of serum creatinine and urea nitrogen at 24 h after IR treatment. Curcumin significantly alleviated renal damage, as serum creatinine and urea nitrogen were markedly lower than those observed in mice in the NC group (Figures 1A,B). Consistent with the serum creatinine and urea nitrogen results, there was a substantial change in the level of histological injury in the IR treated kidneys, with less injury of the tubular epithelial cell in the curcumin group compared with the NC group (Figure 1C). The tubular damage score was markedly decreased in IR-treated kidneys of the curcumin group compared to the NC group (Figure 1D). These data indicate that curcumin may have a nephroprotective effect in mice with IR-induced AKI.

### Curcumin decreases apoptotic cell death

**Figure 2:**
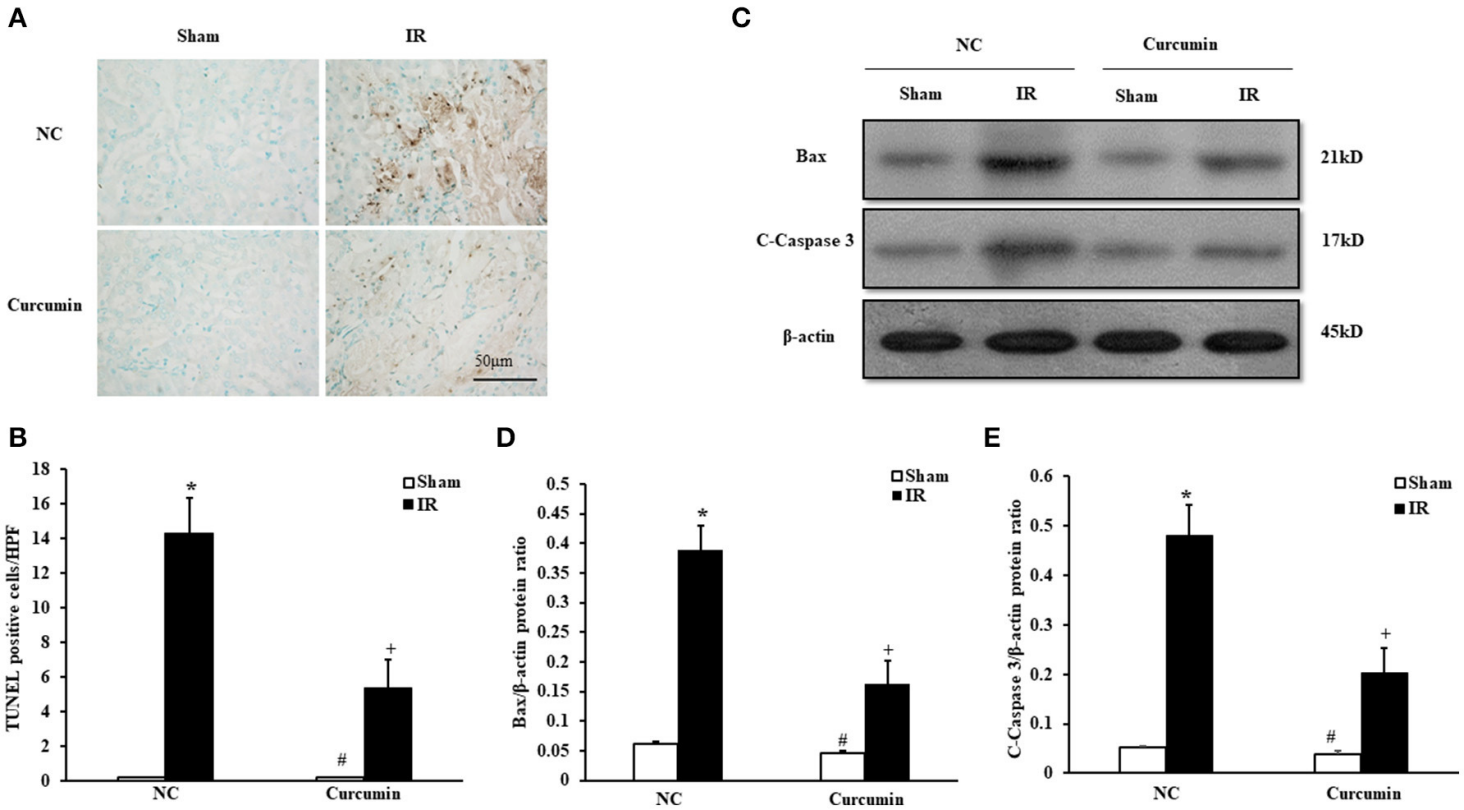
Curcumin decreases apoptotic cell death. **(A)** Representative photomicrographs of kidney sections stained for apoptotic cells (brown) and counterstained with methyl green (green). Scale bar: 50 μm. **(B)** Quantitative analysis of TUNEL-positive cells in the kidneys. ^*^P < 0.05 vs. Sham treatment in the NC group; ^#^*P* < 0.05 vs. IR treatment in the Curcumin group; ^+^*P* < 0.05 vs. IR treatment in the NC group. *n* = 6 in each group. HPF, high power field; TUNEL, terminal transferase dUTP nick-end labeling. **(C)** Representative Western blots show Bax and C-Caspase-3 protein levels in the kidneys after sham or IR. **(D)** Quantitative analysis of Bax protein levels in the kidneys. ^*^P < 0.05 vs. Sham treatment in the NC group; ^#^*P* < 0.05 vs. IR treatment in the Curcumin group; ^+^*P* < 0.05 vs. IR treatment in the NC group. *n* = 6 in each group. **(E)** Quantitative analysis of C-Caspase-3 protein levels in the kidneys. ^*^P < 0.05 vs. Sham treatment in the NC group; ^#^*P* < 0.05 vs. IR treatment in the Curcumin group; ^+^*P* < 0.05 vs. IR treatment in the NC group. *n* = 6 in each group.

A host of evidence has indicated that apoptosis of renal tubular epithelial cells is an important manifestation of IR-induced AKI (Gao et al., 2012). Thus, the extent of apoptosis in IR-treated tubular epithelial cells was detected in both curcumin and NC treated mice. Figure 2A provides the amount of apoptotic tubular cells in kidneys via terminal transferase dUTP nick end labeling (TUNEL) assay. The results showed that IR treatment induced a large number of apoptotic cells in kidneys. TUNEL cells were markedly decreased in IR-treated kidneys of the curcumin group (Figure 2B). Bax and C-caspase-3 have been used to evaluate the level of apoptosis in a number of studies because the two proteins are sensitive to apoptosis. They are the most commonly used indices of apoptosis. Thus, we evaluated apoptosis in kidney tubules cells by Bax and C-caspase-3 protein expression using Western blotting (Figures 2C–E). The results showed that IR treatment led to upregulation of Bax and C-caspase-3 protein expression in the kidneys of mice, while the expression of Bax and C-caspase-3 were markedly decreased in kidneys of mice receiving i.p. curcumin compared to NC treatment. These data indicated that curcumin could reduce apoptosis in kidneys of mice with IR-induced AKI.

### Curcumin increases APPL1 and decreases phosphorylated Akt expression in IR treated mouse kidneys

**Figure 3:**
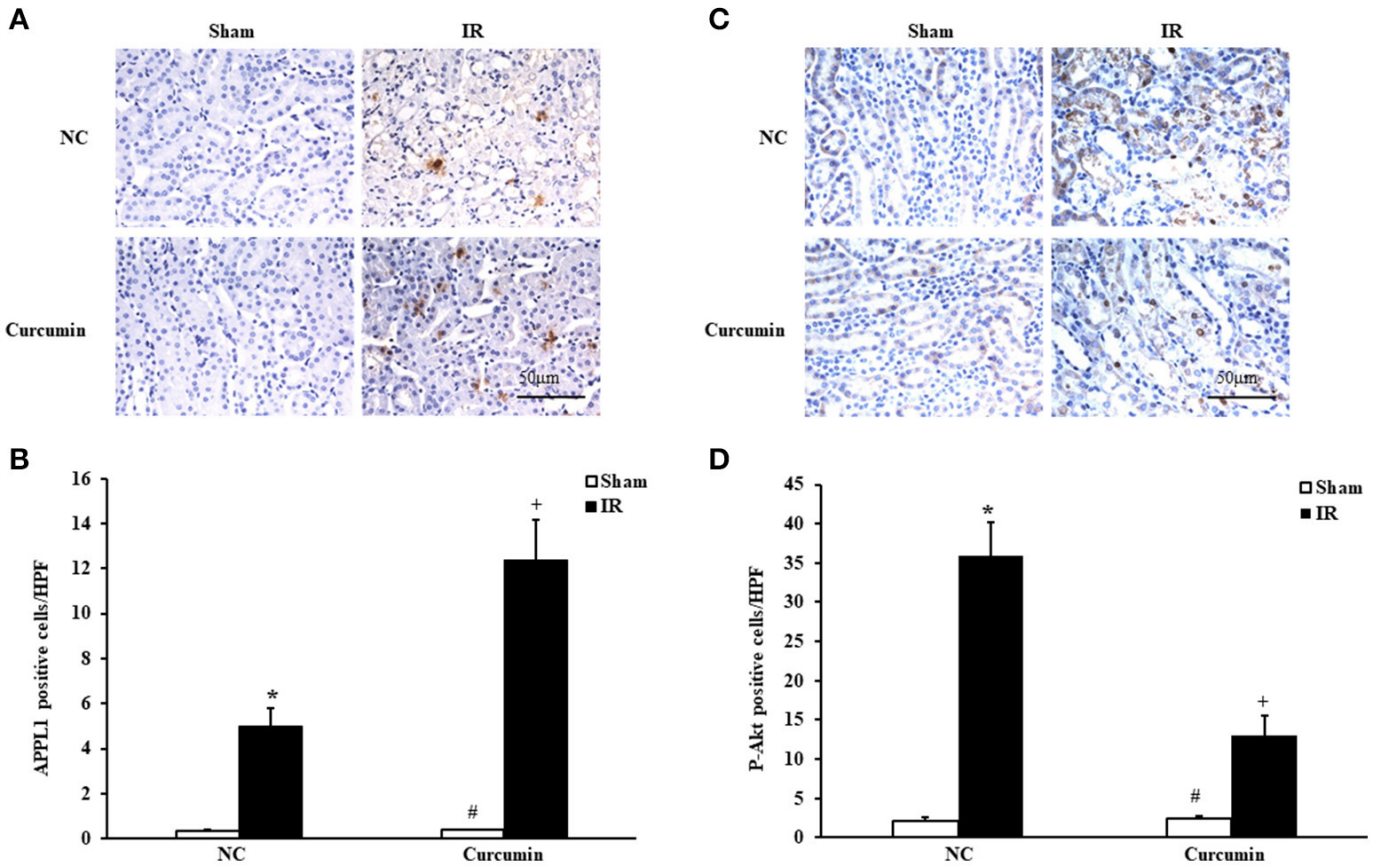
Curcumin increases APPL1 and phosphorylated Akt in IR treated mouse kidneys. **(A)** Representative photomicrographs of kidney sections stained for APPL1 (brown) and counterstained with hematoxylin (blue). Scale bar: 50 μm. **(B)** Quantitative analysis of APPL1 positive cells in the kidneys. ^*^P < 0.05 vs. Sham treatment in the NC group; ^#^*P* < 0.05 vs. IR treatment in the Curcumin group; ^+^*P* < 0.05 vs. IR treatment in the NC group. *n* = 6 in each group. **(C)** Representative photomicrographs of kidney sections stained for phosphorylated Akt (brown) and counterstained with hematoxylin (blue). Scale bar: 50 μm. **(D)** Quantitative analysis of phosphorylated Akt positive cells in the kidneys. ^*^P < 0.05 vs. Sham treatment in the NC group; ^#^*P* < 0.05 vs. IR treatment in the Curcumin group; ^+^*P* < 0.05 vs. IR treatment in the NC group. *n* = 6 in each group.

The role of adiponectin (APN) in ischemia of some important organs has been reported, and the Akt signaling pathway was found to be a downstream signaling pathway of APN involved in a variety of pathological processes (Cheng et al., 2013). Considering that the adaptor protein, phosphotyrosine interaction, pleckstrin homology domain, and leucine zipper containing 1 (APPL1) is an adapter protein that positively mediates adiponectin signaling, we examined the expression of APPL1 and phosphorylated Akt (p-Akt) in kidneys of mice via immunohistochemical staining. Significantly increased APPL1 and p-Akt positive cells were observed in the kidneys of IR-treated mice compared to sham controls. Curcumin treatment significantly increased the expression of APPL1 and inhibited the activation of Akt in the kidneys after IR treatment (Figure 3). These results suggested that APPL1 and the Akt signaling pathway could play an important role in the pathogenesis of IR-induced AKI.

### Expression of APPL1 and phosphorylated Akt in curcumin treated HR model *in vitro*

**Figure 4:**
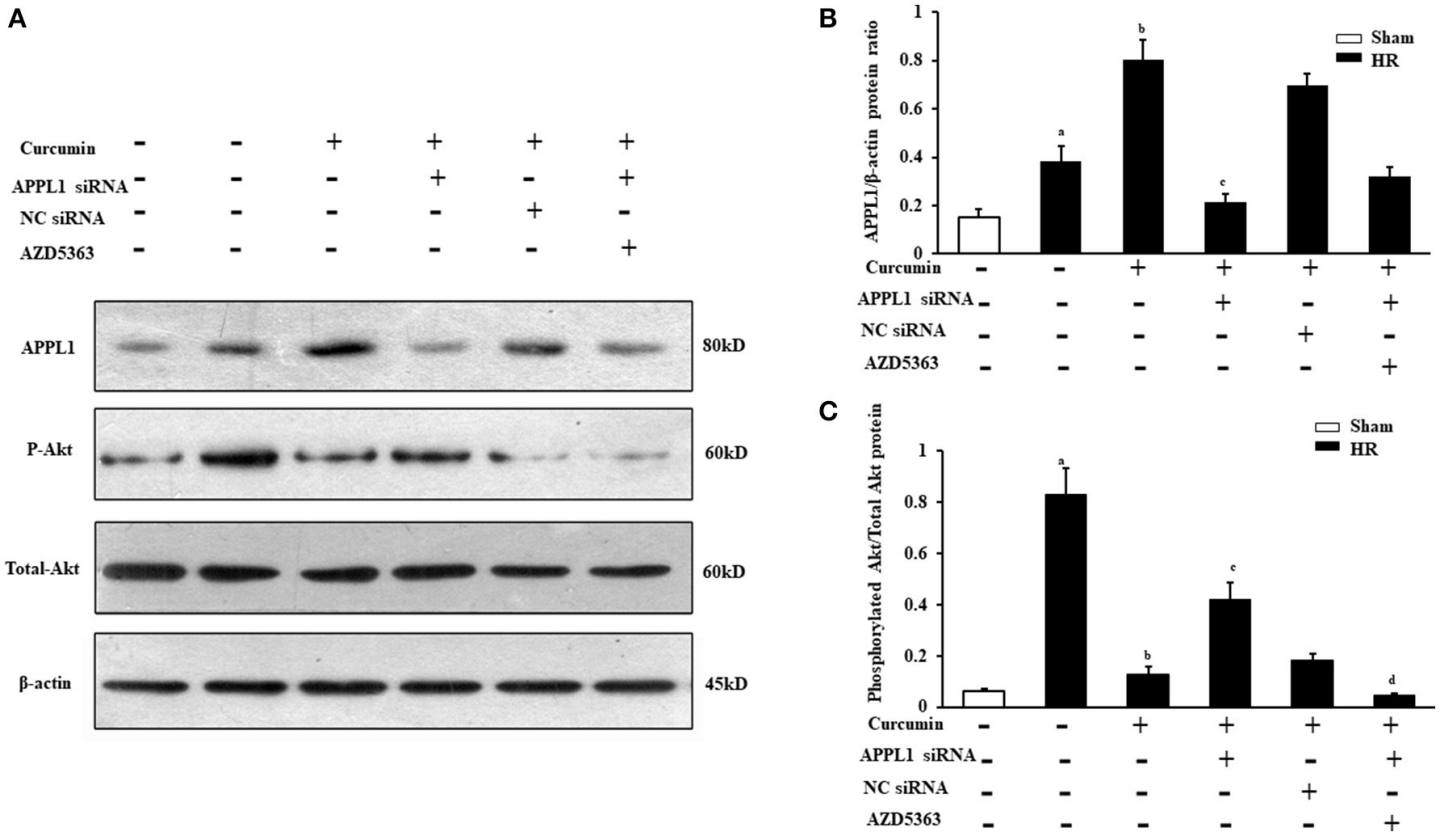
Expression of APPL1 and phosphorylated Akt in an *in vitro* curcumin treated HR model. **(A)** Representative Western blots show APPL1 protein and phosphorylated Akt protein levels in renal tubular epithelial cells of each group. **(B)** Quantitative analysis of APPL1 protein levels in the kidneys. ^a^*P* < 0.05 vs. Sham group; ^b^*P* < 0.05 vs. HR group; ^c^*P* < 0.05 vs. HR+Curcumin group. *n* = 4 in each group. **(C)** Quantitative analysis of phosphorylated Akt protein levels in the kidneys. ^a^*P* < 0.05 vs. Sham group; ^b^*P* < 0.05 vs. HR group; ^c^*P* < 0.05 vs. HR+Curcumin group; ^d^*P* < 0.05 vs. HR+Curcumin+APPL1 siRNA group. *n* = 4 in each group.

To further confirm whether curcumin mediated APPL1/Akt protects against IR-induced AKI by inhibiting apoptosis, the effect on the apoptosis of renal tubular epithelial cell treated with HR *in vitro* with curcumin and the role of the APPL1/Akt signaling pathway were verified. First, we observed the expression of APPL1 and phosphorylated Akt via Western blot in our curcumin treated HR model of renal tubular epithelial cells (Figures 4A,C). The results showed that HR induced increased expression of APPL1 and activation of Akt; curcumin treatment significantly upregulated the expression of APPL1 and inhibited the activation of Akt. Downregulation of APPL1 gene expression could directly result in the activation of Akt, while AZD5363 could specifically inhibit Akt phosphorylation (Figures 4B,C).

### Curcumin mediated APPL1/Akt signaling inhibits apoptosis of renal tubular epithelial cells treated with HR

**Figure 5:**
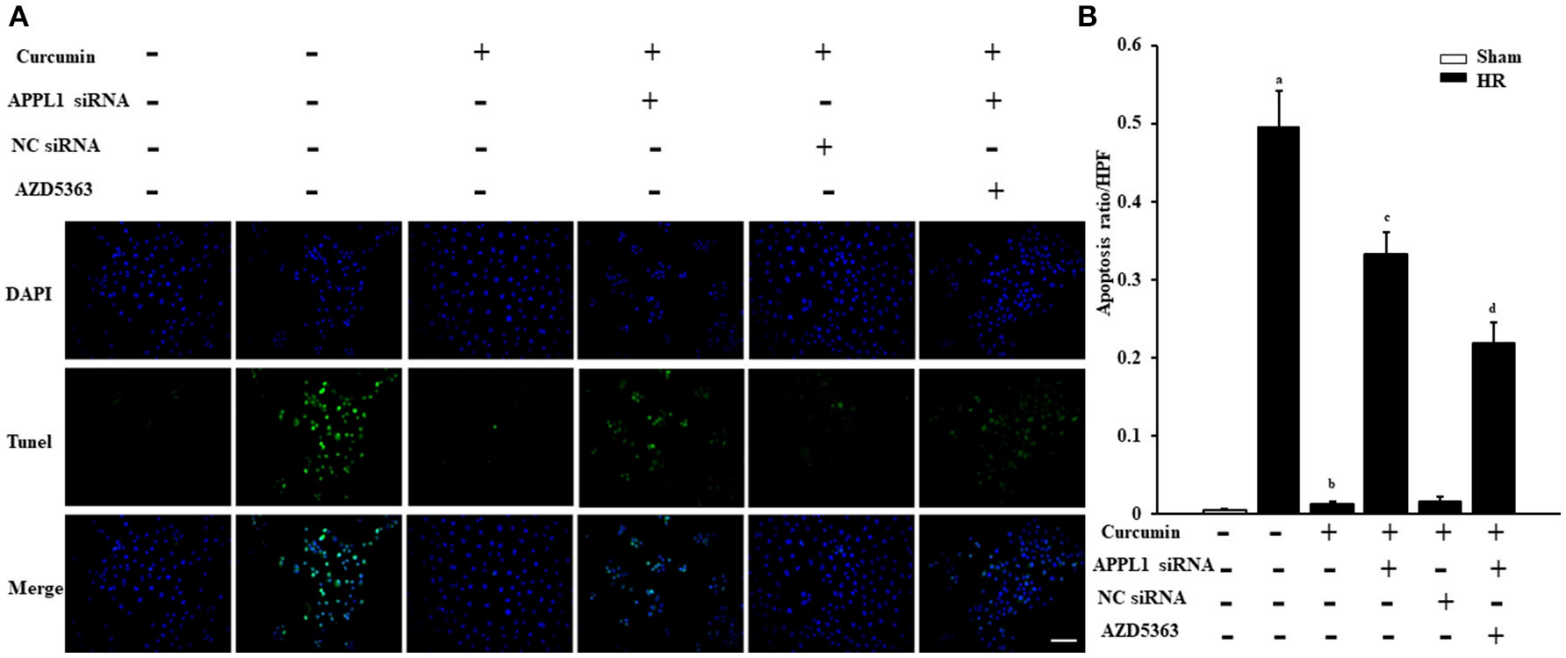
The effect of curcumin on TUNEL positive renal tubular epithelial cells treated with HR. **(A)** TUNEL apoptosis assay in mouse renal tubular epithelial cells. Scale bar: 100 μm. **(B)** Quantitative analysis of the apoptosis ratio of renal tubular epithelial cells in each group. ^a^*P* < 0.05 vs. Sham group; ^b^*P* < 0.05 vs. HR group; ^c^*P* < 0.05 vs. HR+Curcumin group; ^d^*P* < 0.05 vs. HR+Curcumin+APPL1 siRNA group. *n* = 4 in each group.

**Figure 6:**
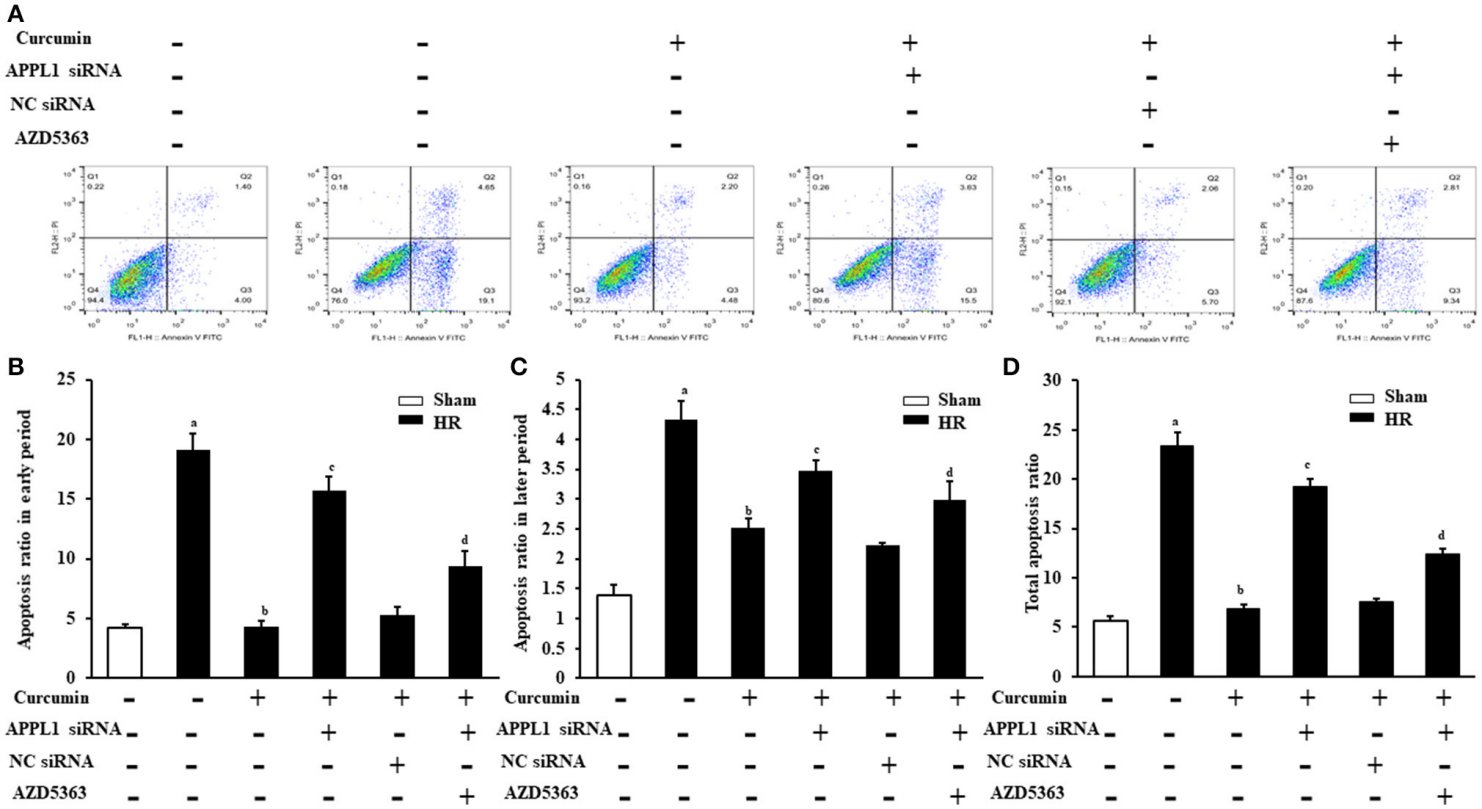
Flow cytometric analysis of the apoptosis rate of renal tubular epithelial cells in each group. **(A)** Flow cytometric analysis of the apoptosis rate of mouse renal tubular epithelial cells. **(B)** The apoptosis rate of renal tubular epithelial cells in each group in the early period. ^a^*P* < 0.05 vs. Sham group; ^b^*P* < 0.05 vs. HR group; ^c^*P* < 0.05 vs. HR+Curcumin group; ^d^*P* < 0.05 vs. HR+Curcumin+APPL1 siRNA group. *n* = 4 in each group. **(C)** The apoptosis rate of renal tubular epithelial cells in each group in the later period. ^a^*P* < 0.05 vs. Sham group; ^b^*P* < 0.05 vs. HR group; ^c^*P* < 0.05 vs. HR+Curcumin group; ^d^*P* < 0.05 vs. HR+Curcumin+APPL1 siRNA group. *n* = 4 in each group. **(D)** The total apoptosis rate of renal tubular epithelial cells in each group in the later period. ^a^*P* < 0.05 vs. Sham group; ^b^*P* < 0.05 vs. HR group; ^c^*P* < 0.05 vs. HR+Curcumin group; ^d^*P* < 0.05 vs. HR+Curcumin+APPL1 siRNA group. *n* = 4 in each group.

The effect on TUNEL cells treated with HR with curcumin is presented in Figure 5A. Our results indicated that TUNEL cells induced by HR were significantly increased and that curcumin treatment can significantly alleviate TUNEL cell induction by HR. In addition, APPL1 siRNA could weaken the inhibition of apoptosis induced by curcumin, while inhibition of Akt could decrease the effect of APPL1 siRNA (Figure 5B). The effect on HR-treated renal tubular epithelial cells administered curcumin using flow cytometry analysis was demonstrated in Figure 6A. The apoptosis rates during the early period (Figure 6B), later period (Figure 6C), and the total period are shown (Figure 6D). In agreement with the TUNEL staining, the results of flow cytometry analysis indicated that curcumin augmented APPL1/Akt signaling to inhibit the apoptosis of renal tubular epithelial cells treated with HR.

**Figure 7:**
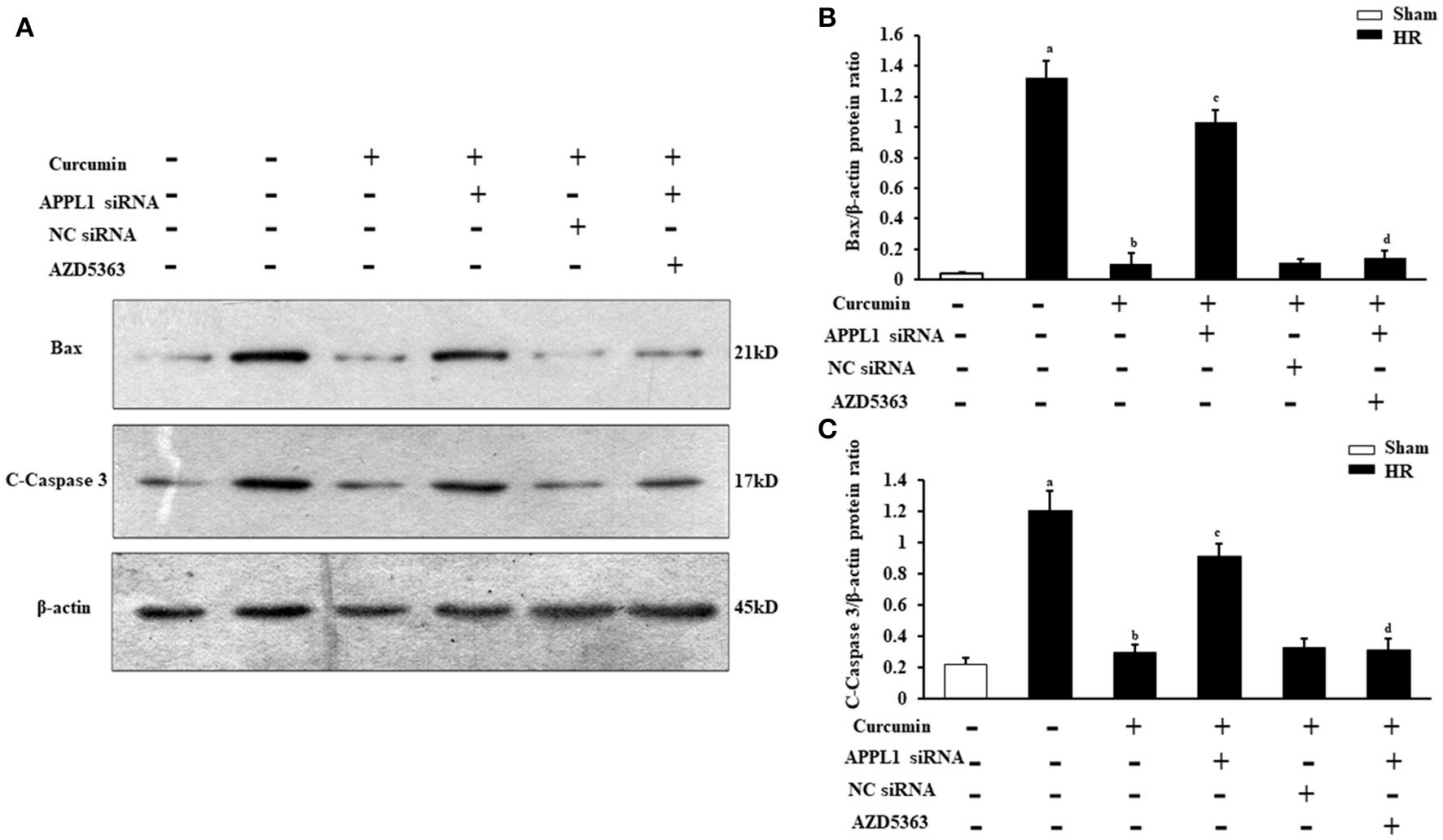
The expression of Bax and C-Caspase-3 in renal tubular epithelial cells in each group. **(A)** Representative Western blots show Bax and C-Caspase-3 protein levels of renal tubular epithelial cells in each group. **(B)** Quantitative analysis of Bax protein levels in renal tubular epithelial cells. ^a^*P* < 0.05 vs. Sham group; ^b^*P* < 0.05 vs. HR group; ^c^*P* < 0.05 vs. HR+Curcumin group; ^d^*P* < 0.05 vs. HR+Curcumin+APPL1 siRNA group. *n* = 4 in each group. **(C)** Quantitative analysis of C-Caspase-3 protein levels in renal tubular epithelial cells. ^a^*P* < 0.05 vs. Sham group; ^b^*P* < 0.05 vs. HR group; ^c^*P* < 0.05 vs. HR+Curcumin group; ^d^*P* < 0.05 vs. HR+Curcumin+APPL1 siRNA group. *n* = 4 in each group.

To provide additional supporting evidence, we also evaluated the effect on the apoptosis of renal tubular epithelial cells treated with IR and curcumin by assessing Bax and C-caspase-3 protein expression via Western blot (Figures 7A,C). The results showed that Bax and C-caspase-3 protein expression were elevated in renal tubular epithelial cells treated with HR, while Bax and C-caspase-3 protein expression were markedly decreased in renal tubular epithelial cells treated with curcumin. Downregulation of APPL1 expression could reduce the inhibition of apoptosis with curcumin treatment, and inhibition of Akt could weaken the effect of APPL1 siRNA (Figures 7B,C). All the original pictures can be seen in the Supplementary Material.

## Discussion

IR induced-AKI is a clinical syndrome characterized by rapid kidney dysfunction and high mortality (Barkhordari et al., 2011; Malek and Nematbakhsh, 2015), and has thus been of great interest to both clinicians and academics. Treatment strategies or therapeutics that prevent or reduce IR-induced AKI have important clinical significance. Curcumin is a yellow spice derived from the roots (rhizomes) of *Curcuma longa*, commonly known as turmeric, whose principal chemical ingredient was identified as (1E, 6E)-1,7-bis (4-hydroxy- 3-methoxyphenyl)-1,6-heptadiene-3,5-dione or diferuloylmethane (Rahman et al., 2006; Chakraborty et al., 2014). As a non-toxic natural product, curcumin was traditionally used alone or in combination as an anti-inflammatory, antioxidant, anti-carcinogenic, and antimicrobial agent in oriental cultures and has been scientifically proven to have antioxidant, anti-inflammatory and antibacterial properties (Calabrese et al., 2008; Aggarwal and Harikumar, 2009; Augustyniak et al., 2010). In recent years, mounting evidence has indicated that curcumin has a protective effect on multiple organs, including hepatoprotective, neuroprotective, cardioprotective, and renoprotective effects (Osawa, 2007; Reyes-Fermín et al., 2012; Soetikno et al., 2013a; Avci et al., 2017). In the present study, the renoprotective effect of curcumin in a mice model of IR-induced AKI was demonstrated. Our results also indicated that curcumin mediates the upregulation of APPL1 to protect against IR-induced AKI by inhibiting Akt phosphorylation.

Although numerous studies have suggesting that curcumin is a promising renoprotective reagent against AKI, most previous efforts have been spent on improving the renoprotective effect of curcumin with renal injury induced by drugs and chemicals (Antunes et al., 2001; Eybl et al., 2004; Tirkey et al., 2005; Manikandan et al., 2011). Moreover, the renoprotective effect of curcumin on diabetic nephropathy (DN) has also been researched (Soetikno et al., 2012, 2013b). In fact, the effect of curcumin on AKI induced by I/R has also been studied. Curcumin significantly attenuated the reduction of serum GPx and the levels of urea, cystatin C, and MDA in the serum and increased the concentrations of MDA, nitric oxide, and protein carbonyls in rat kidneys with I/R (Ullah et al., 2017). In our study, we investigated the effect of curcumin on the kidneys of mice treated with IR using serum creatine, BUN, and pathological injury. Our study showed that curcumin alleviated renal damage in the pathogenesis of IR-induced AKI.

Recently, there has been a substantial amount of research that explored the pharmacological function of curcumin, but the precise mechanisms of these effects are not fully understood. Curcumin is known to have multiple therapeutic actions, including anti-inflammatory, anti-microbial, anti-oxidant, anti-cancer, etc. (Deng et al., 2016; Rezaee et al., 2017). Curcumin provides cytoprotection via anti-inflammatory and multiple antioxidant mechanisms following renal IR injury (Rogers et al., 2012). Moreover, the mechanism of apoptosis plays an important role in AKI and is also involved in the effect of curcumin. Interestingly, some studies have reported that curcumin can induce apoptosis through the mitochondrial pathway (Zhang et al., 2017), while other results showed that curcumin can play a protective role after I/R injury by inhibiting apoptosis (Wang L., et al., 2017; Wang S., et al., 2017). However, the effect on apoptosis following treatment with curcumin in the pathogenesis of IR-induced AKI has not been fully elucidated. Our data indicated that renal tubular epithelial cell apoptosis was markedly increased by IR and that curcumin could reduce kidney apoptosis in mice with IR-induced AKI.

As a serine/threonine kinase, Akt/protein kinase B (PKB) acts as a key node in diverse signaling cascades in both normal cellular physiology and during various disease states. Akt interacts with complicated signaling targets to regulate multiple biological processes, including apoptosis, autophagy, cell cycle progression, etc. (Ni et al., 2014; Han et al., 2017). A large number of evidences have proved that Akt signaling pathway mediates acute and chronic injury of several important organs. Hingtgen SD has demonstrated that inactivation of Akt by dominant-negative Akt inhibits cardiomyocyte hypertrophy *in vitro* (Hingtgen et al., 2010). Zhu et al. directly indicated that suppressing of the PI3K/Akt was involved in the protective effect of catalpol on renal ischemia/reperfusion-injury (Zhu et al., 2015). Chen et.al showed that the protection of renal injury in diabetic rats is related to the inhibition of the PI3K/AKT pathway (Chen et al., 2017). However, the studies with Xie (Xie et al., 2017) and Kalmar-Nagy (Kalmar-Nagy et al., 2013) have shown that alleviating renal injury is associated with activation of the AKT signaling pathway. So the role of AKT signaling pathway is controversial in the current research. The possible reason is that the phosphorylation sites of Akt include Thr308 and Ser473, and Akt has three subtypes of Akt1, Akt2, and Akt3. In this study, the phosphorylation site of AKT we selected is Ser473, and the phosphorylation site of Akt selected by these two literatures may be different from ours. In fact, in our experiment we found an interesting phenomenon the expression trend of the two phosphorylation sites of Akt is the opposite in the AKI model. Of course, it needs more verification and further research. In addition, different detection methods and experimental conditions may also result in different conclusions. Moreover, Akt is a common signaling pathway regulating apoptosis and autophagy, and is also regulated by many other factors, so it has different states in different pathophysiological changes. Therefore, the role of Akt signaling pathway may exert different effects under different pathological conditions, and this requires us to further study in the future. In any case, this study demonstrated that curcumin activates APPL1 and inhibits Akt phosphorylation and alleviates AKI.

Furthermore, as a crucial element of protein trafficking and cell signaling, APPL1 can mediate the Akt signaling pathway to augment various pathophysiological processes (Tan et al., 2016; Wang et al., 2016). Thus, based on our APPL1 and phosphorylated Akt results, we investigated whether the APPL1/Akt signaling pathway mediated the effect of curcumin on IR-induced AKI. Our data indicated that curcumin may upregulate the expression of APPL1 and inhibit Akt signaling pathway activation to inhibit apoptosis and protect against IR-induced AKI. AZD5363, a novel AKT inhibitor, could specifically inhibit Akt phosphorylation. Our data indicated that the expression of APPL1 was increased after renal tubular epithelial cells treatment with AZD5363, which may offset the effect of APPL1 siRNA. Thus, the AZD5363 treatment has a partial effect in reversing apoptosis of renal tubular epithelial cells apoptosis after treatment with curcumin+APPL1 siRNA. Akt pathway can mediate cell growth and survival in many cell types (Hausenloy et al., 2005) and more recently has been implicated in the protection of kidney against ischemia/reperfusion-induced injury by moderating the inflammatory response (Sano et al., 2010; Ravingerova et al., 2012). The pathophysiological mechanism of I/R injury includes endothelial dysfunction, oxidative stress and pro-inflammatory cytokines and activation of apoptotic pathways (Munshi et al., 2011). Rogers NM demonstrated that curcumin provides cytoprotection via anti-inflammatory and multiple antioxidant mechanisms following renal IR injury (Rogers et al., 2012). Our results have shown that curcumin decreases phosphorylated Akt expression in IR treated mouse kidneys. Thus, we speculate that curcumin may inhibit phosphorylation of Akt to protect against ischemia/reperfusion-induced AKI.

In summary, our results found that curcumin exhibited a protective effect against IR-induced AKI via upregulation of APPL1 that subsequently inhibits Akt signaling pathway activation. Thus, curcumin may be a potential treatment for IR-induced AKI, and APPL1/Akt may be a major therapeutic signaling pathway underlying the nephroprotective effect of curcumin.

## Author contributions

JuZ and YF are responsible for the design and implementation of all the experiments. HP and FH are responsible for completing the animal experiment. JiZ and HC are responsible for completing the cell experiment.

### Conflict of interest statement

The authors declare that the research was conducted in the absence of any commercial or financial relationships that could be construed as a potential conflict of interest. The reviewers AS, VP and handling Editor declared their shared affiliation.
