# Supplementary figures and images for: Molecular Mechanisms of Curcumin Renoprotection in Experimental Acute Renal Injury
Source: Front Pharmacol. 2017 Dec 12;8:912. doi: 10.3389/fphar.2017.00912 (PMC5733093; doi:10.3389/fphar.2017.00912)

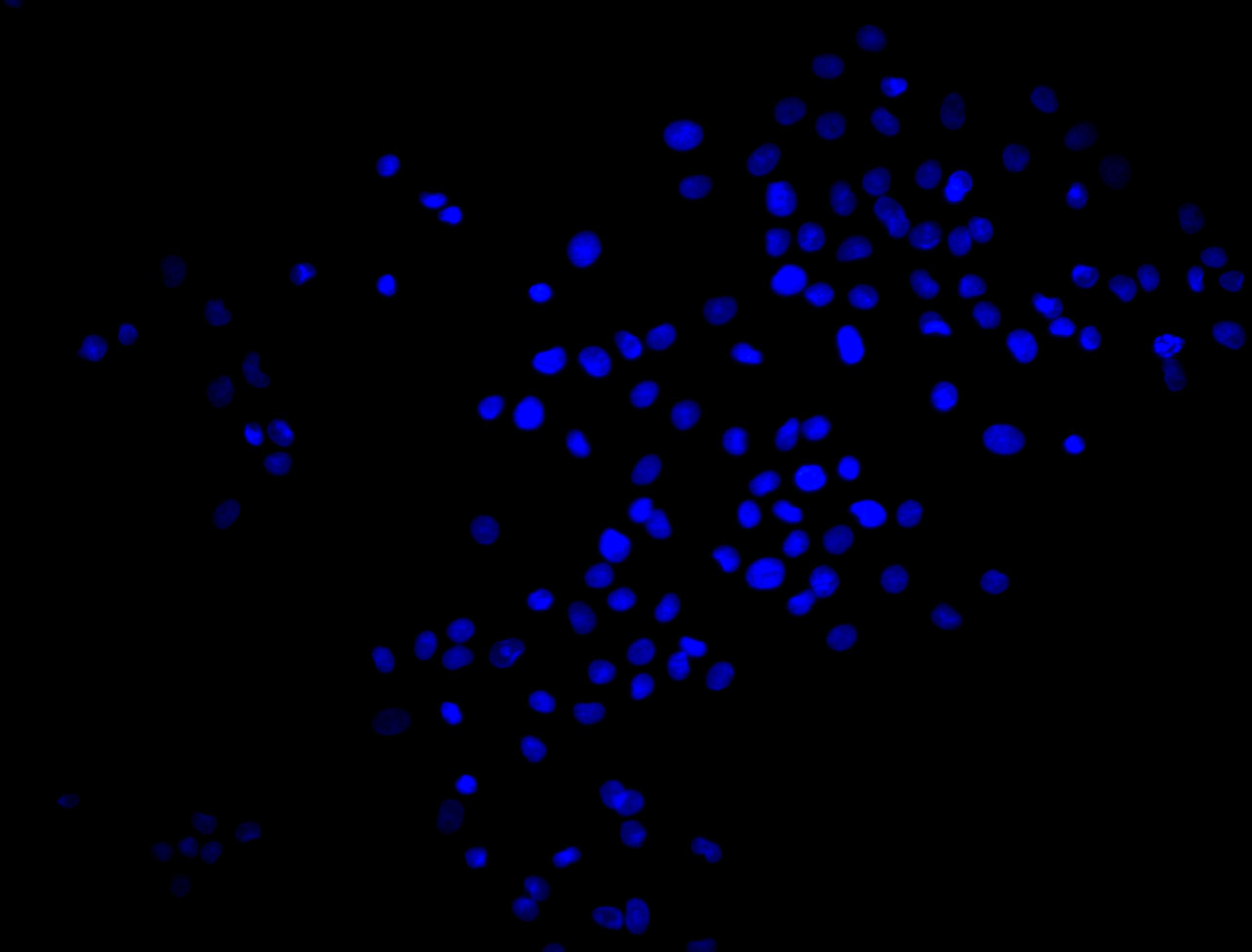

Supplement: Supplementary file 1 [file Presentation1.zip › original images-1/azd (4).jpg]

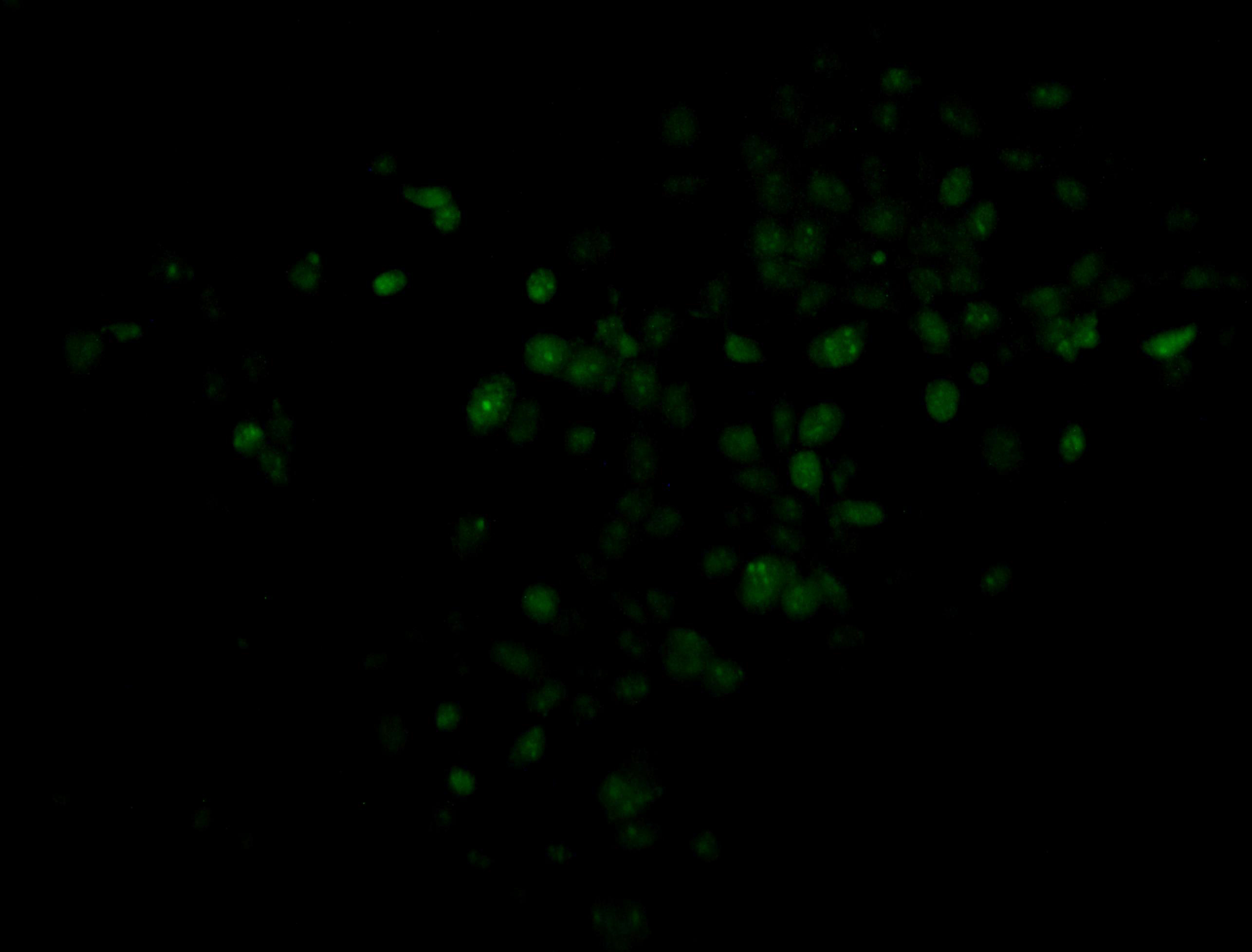

Supplement: Supplementary file 1 [file Presentation1.zip › original images-1/azd (5).jpg]

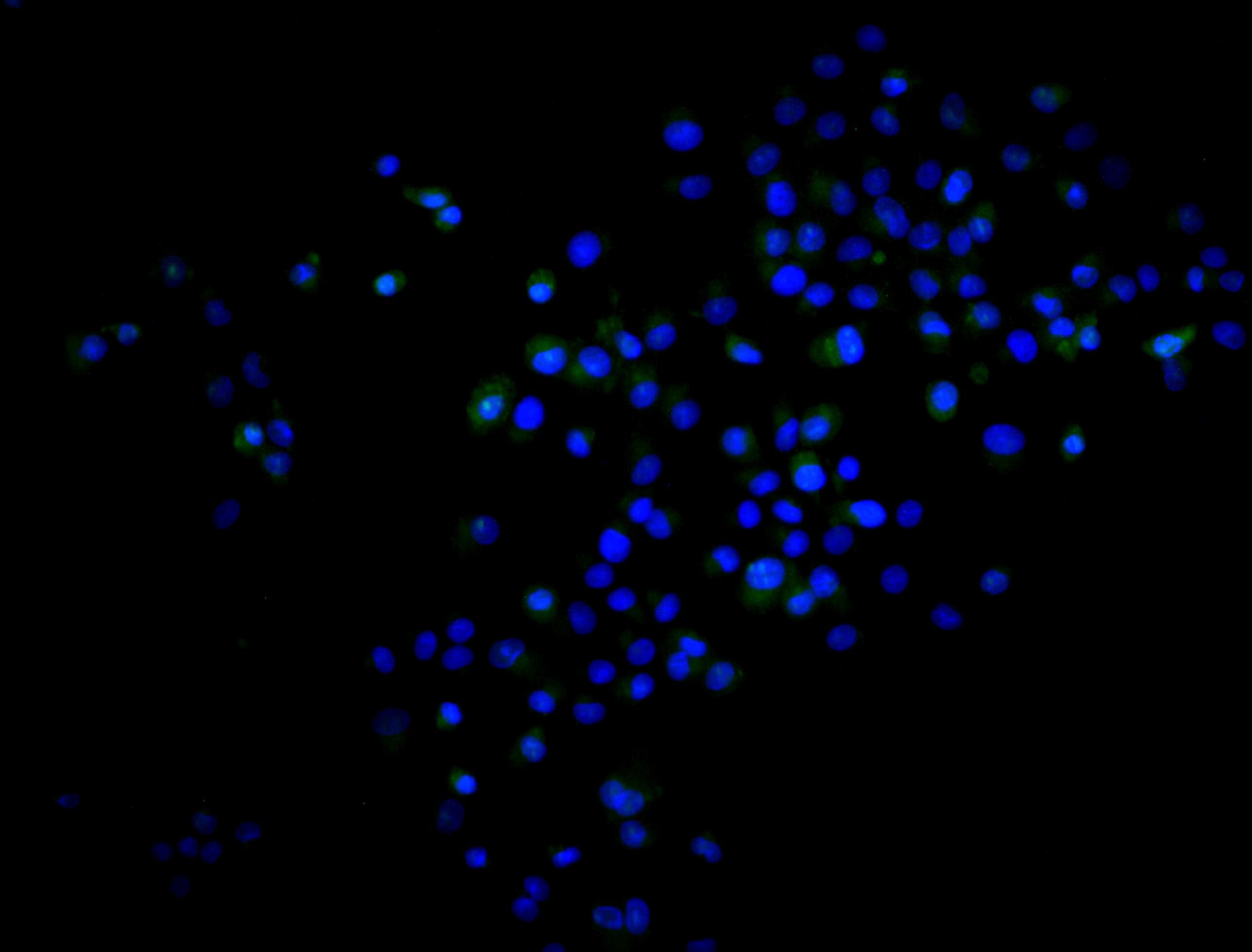

Supplement: Supplementary file 1 [file Presentation1.zip › original images-1/azd (6).jpg]

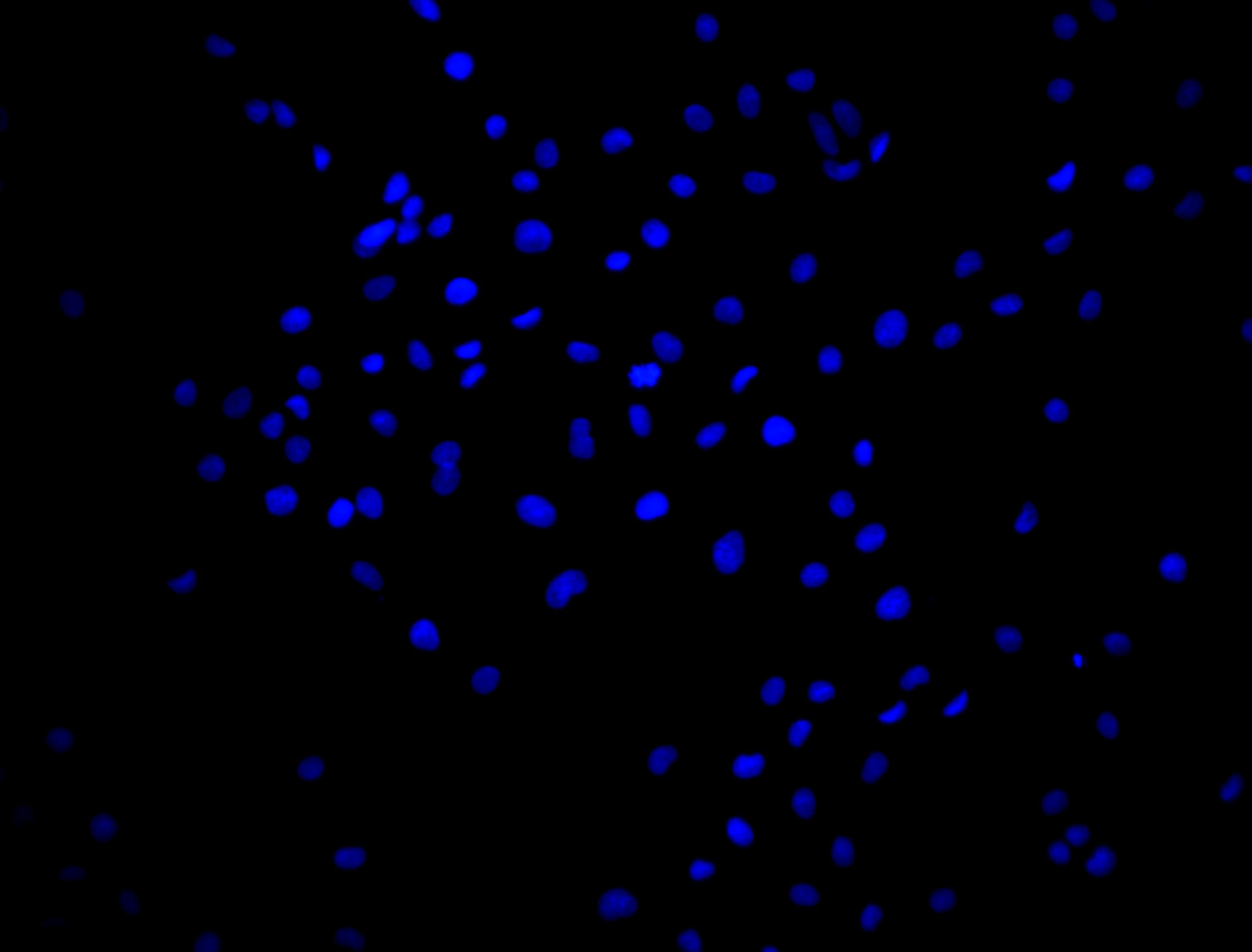

Supplement: Supplementary file 1 [file Presentation1.zip › original images-1/cell (4).jpg]

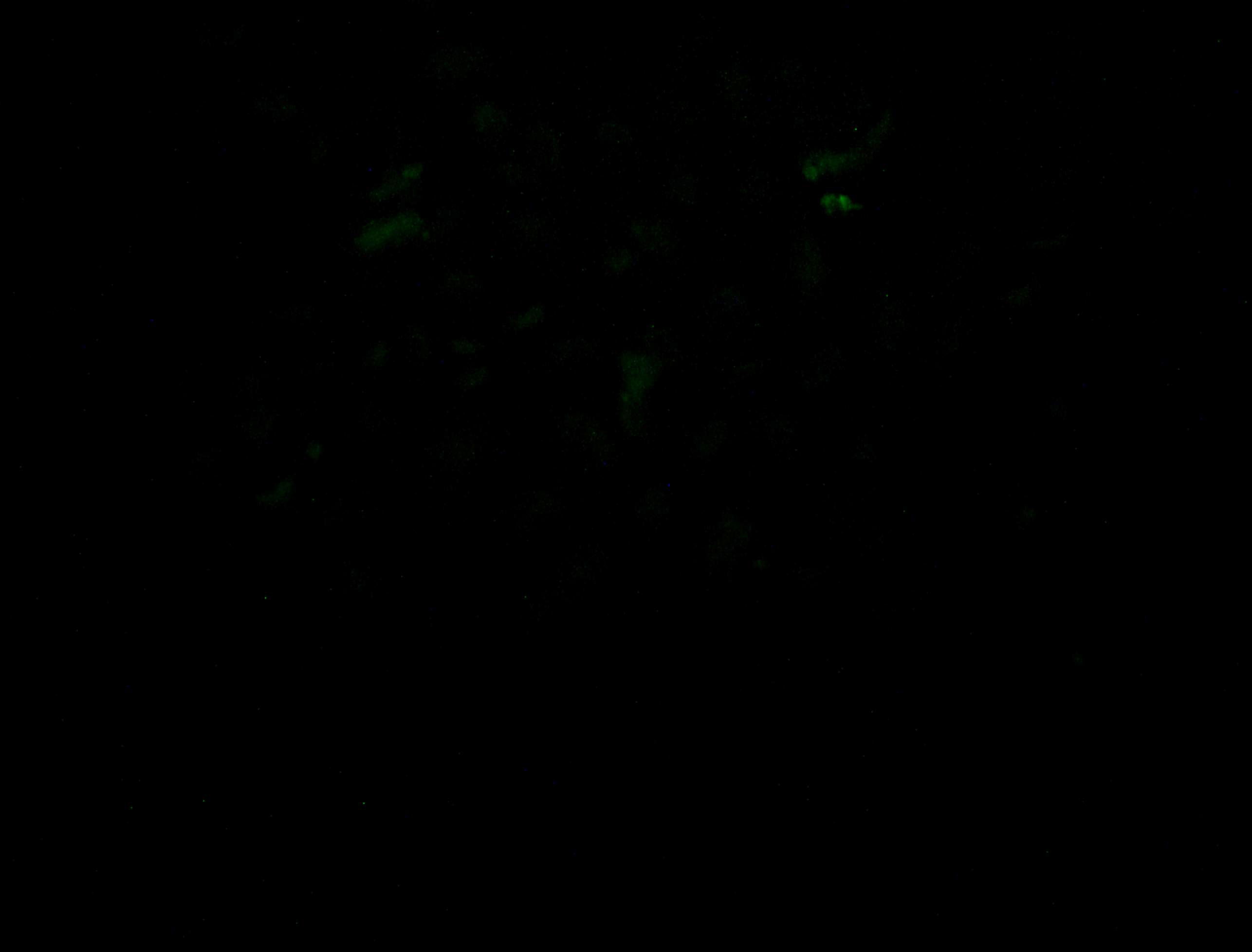

Supplement: Supplementary file 1 [file Presentation1.zip › original images-1/cell (5).jpg]

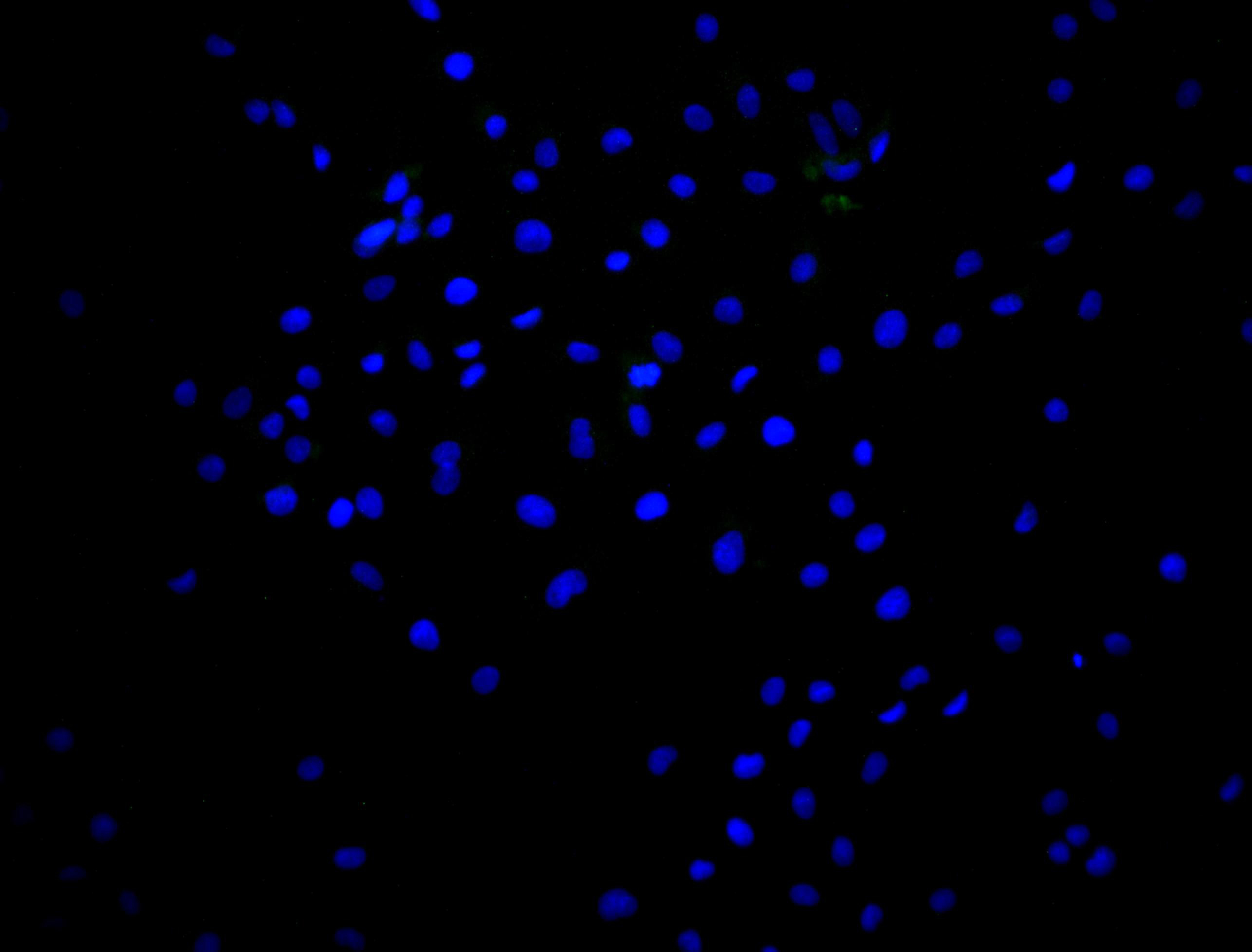

Supplement: Supplementary file 1 [file Presentation1.zip › original images-1/cell (6).jpg]

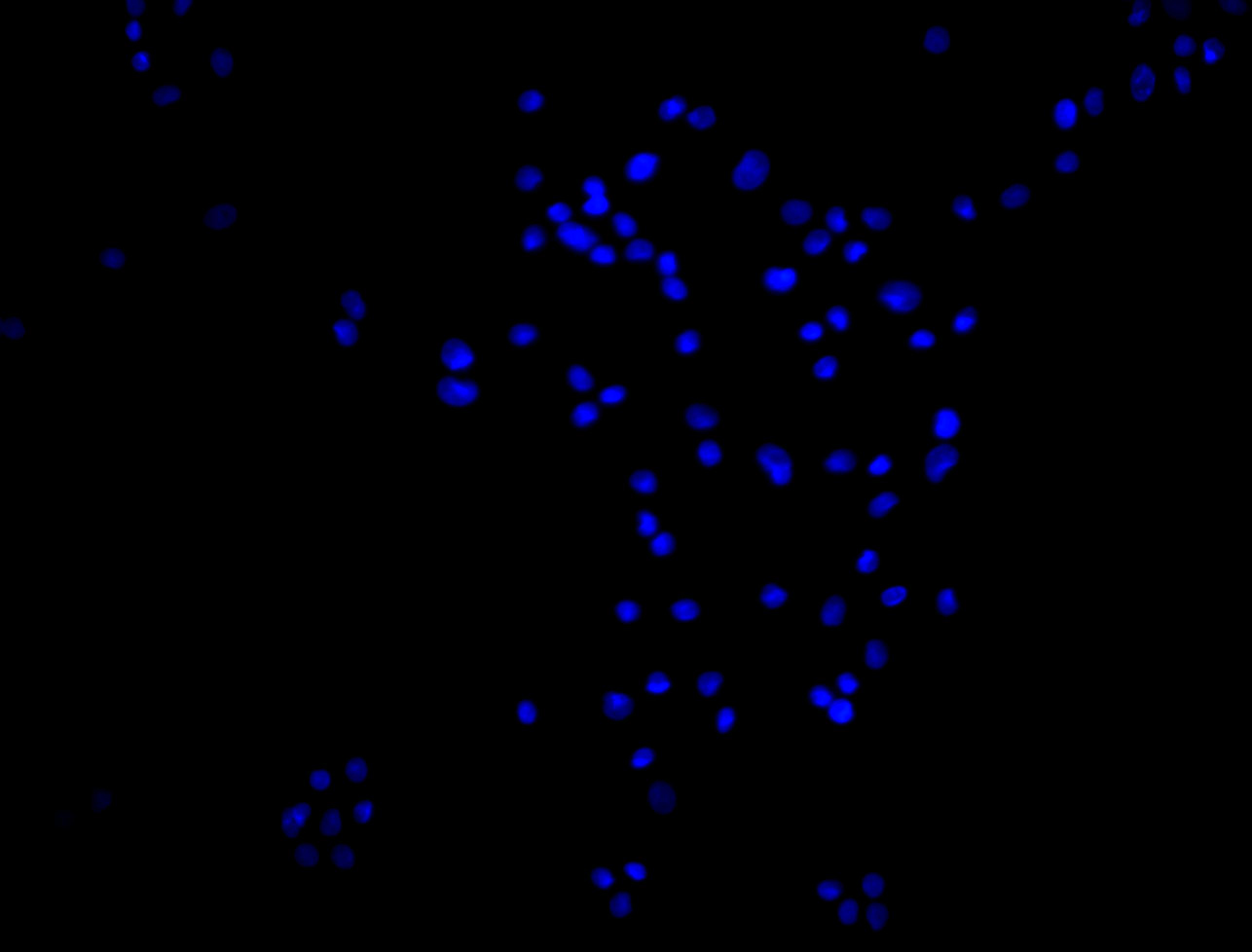

Supplement: Supplementary file 1 [file Presentation1.zip › original images-1/hr (7).jpg]

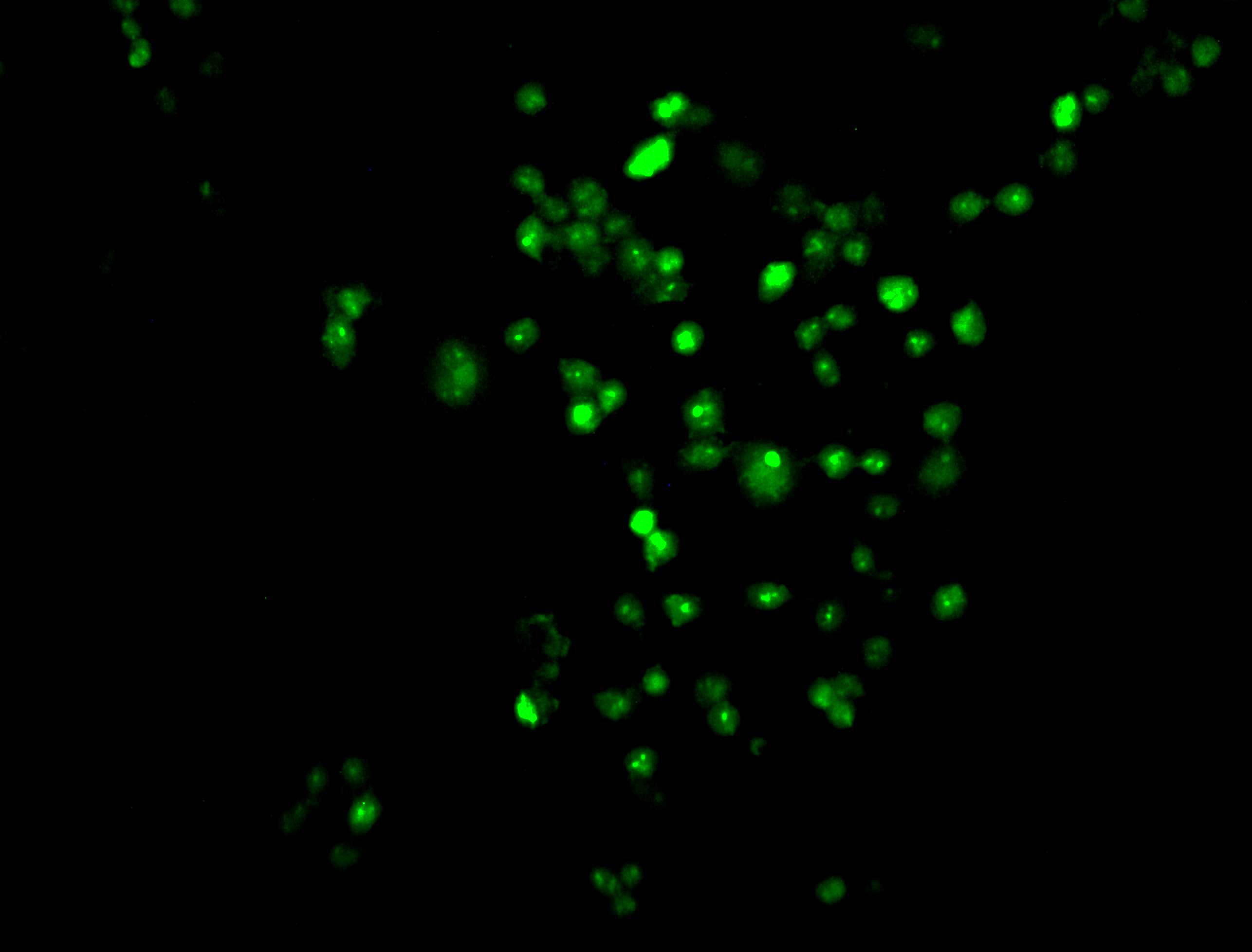

Supplement: Supplementary file 1 [file Presentation1.zip › original images-1/hr (8).jpg]

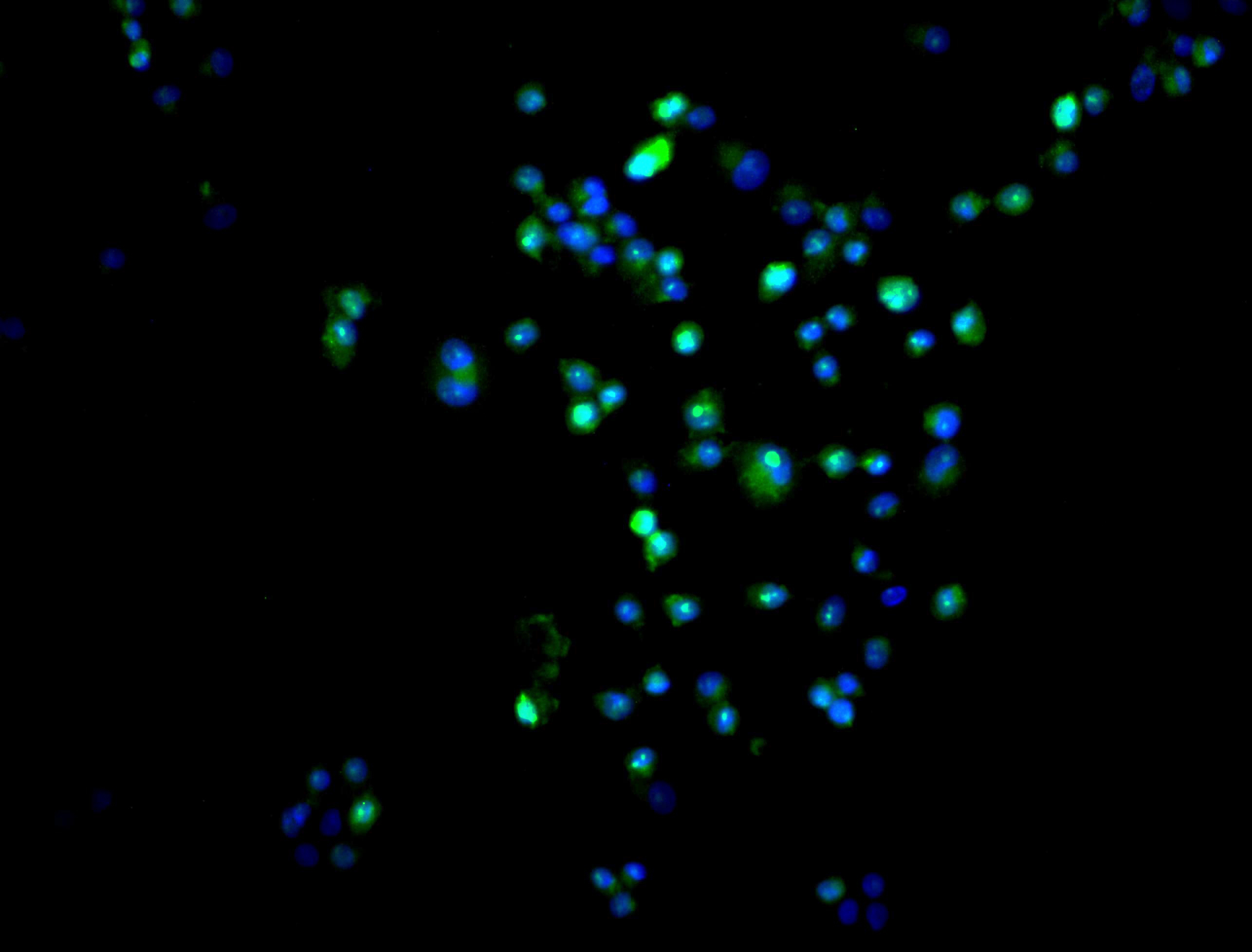

Supplement: Supplementary file 1 [file Presentation1.zip › original images-1/hr (9).jpg]

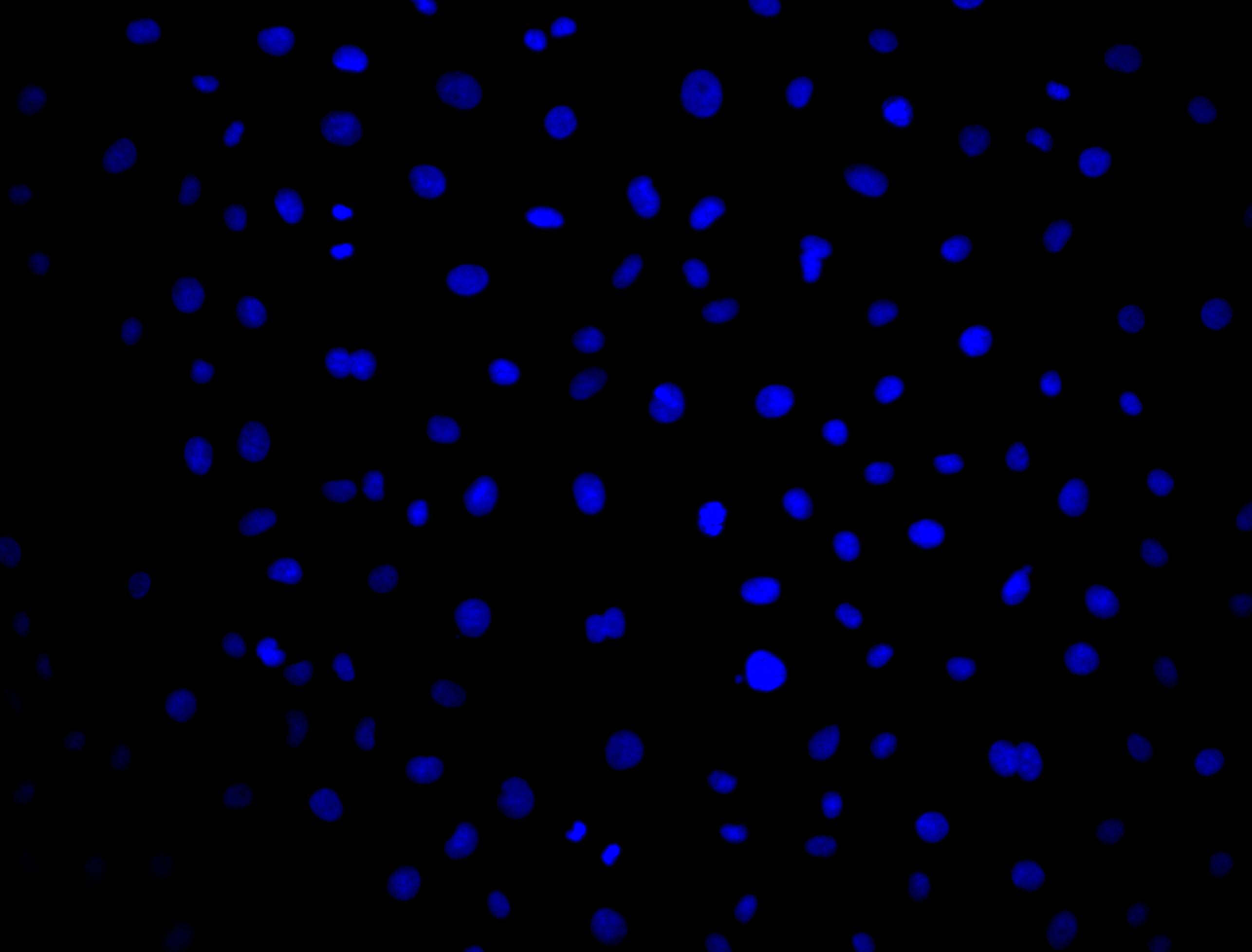

Supplement: Supplementary file 1 [file Presentation1.zip › original images-1/hr+cur (7).jpg]

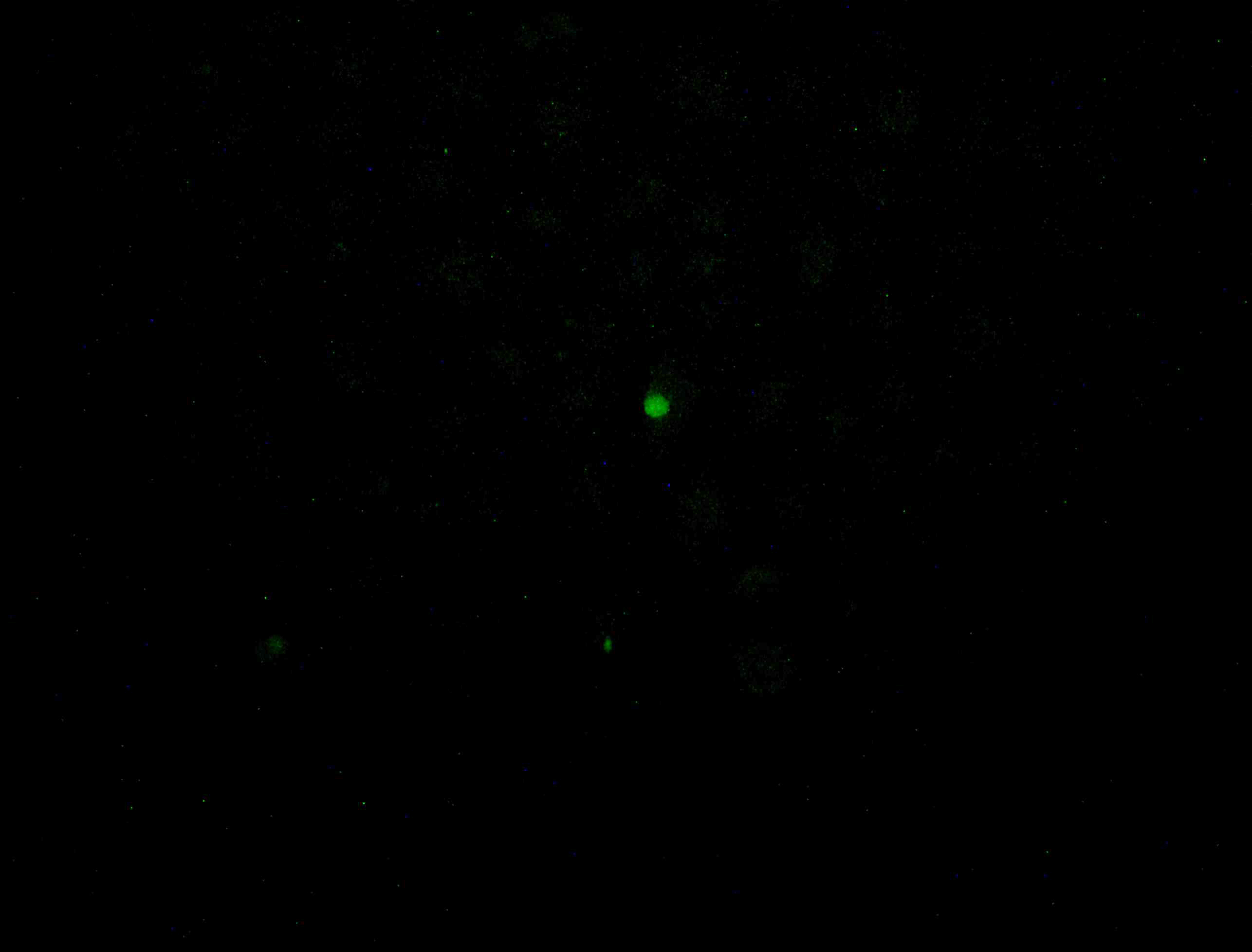

Supplement: Supplementary file 1 [file Presentation1.zip › original images-1/hr+cur (8).jpg]

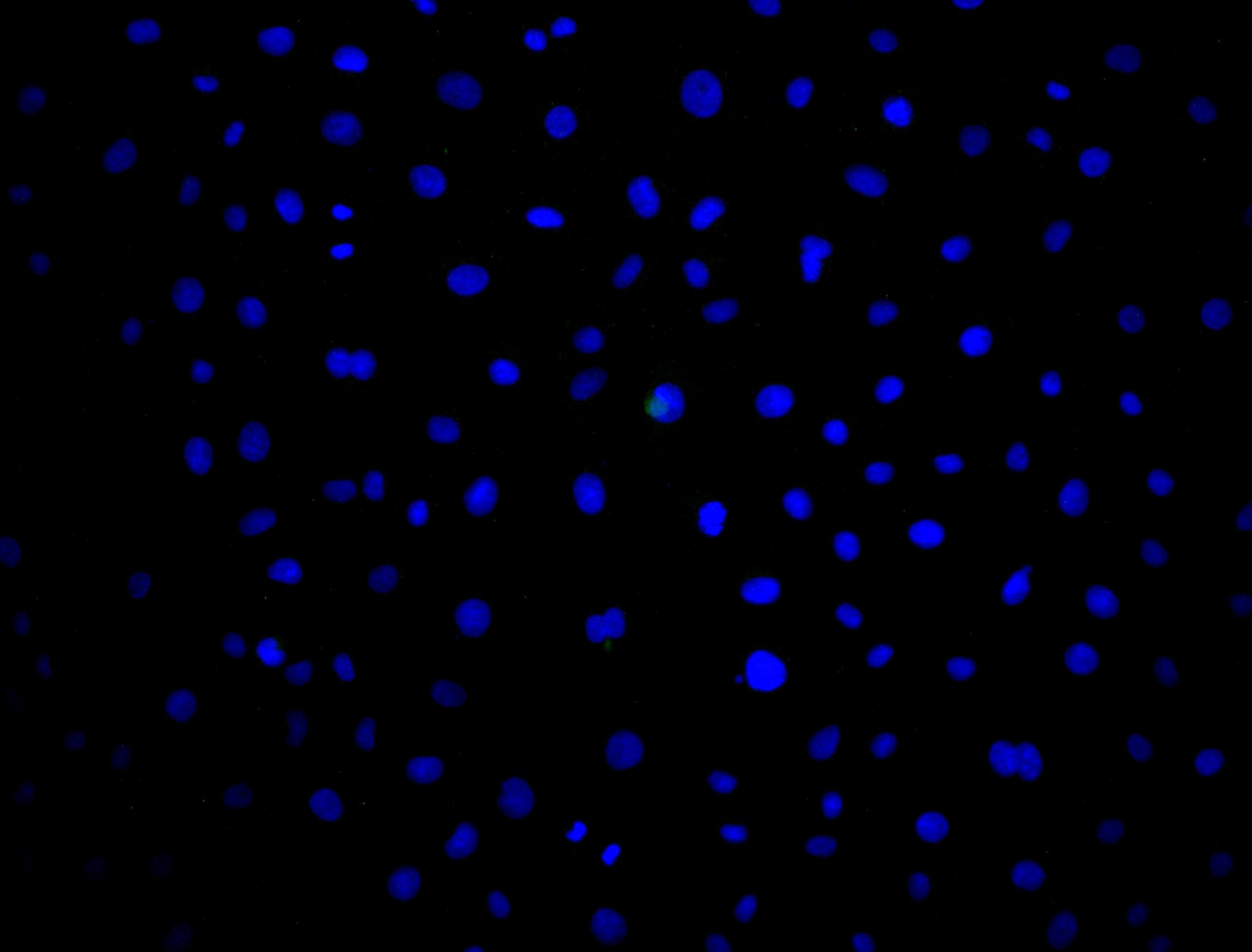

Supplement: Supplementary file 1 [file Presentation1.zip › original images-1/hr+cur (9).jpg]

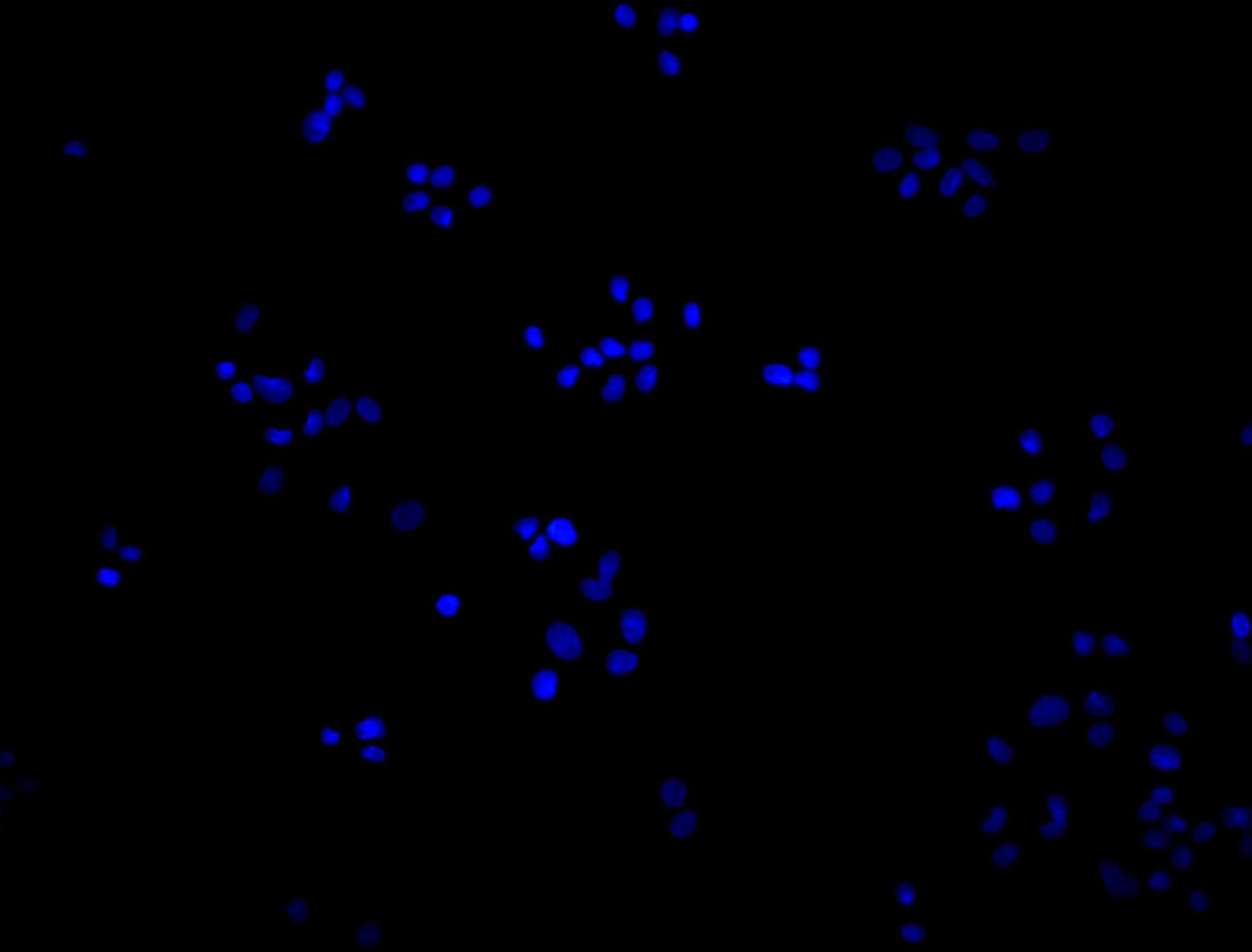

Supplement: Supplementary file 1 [file Presentation1.zip › original images-1/hr+cur+asi (7).jpg]

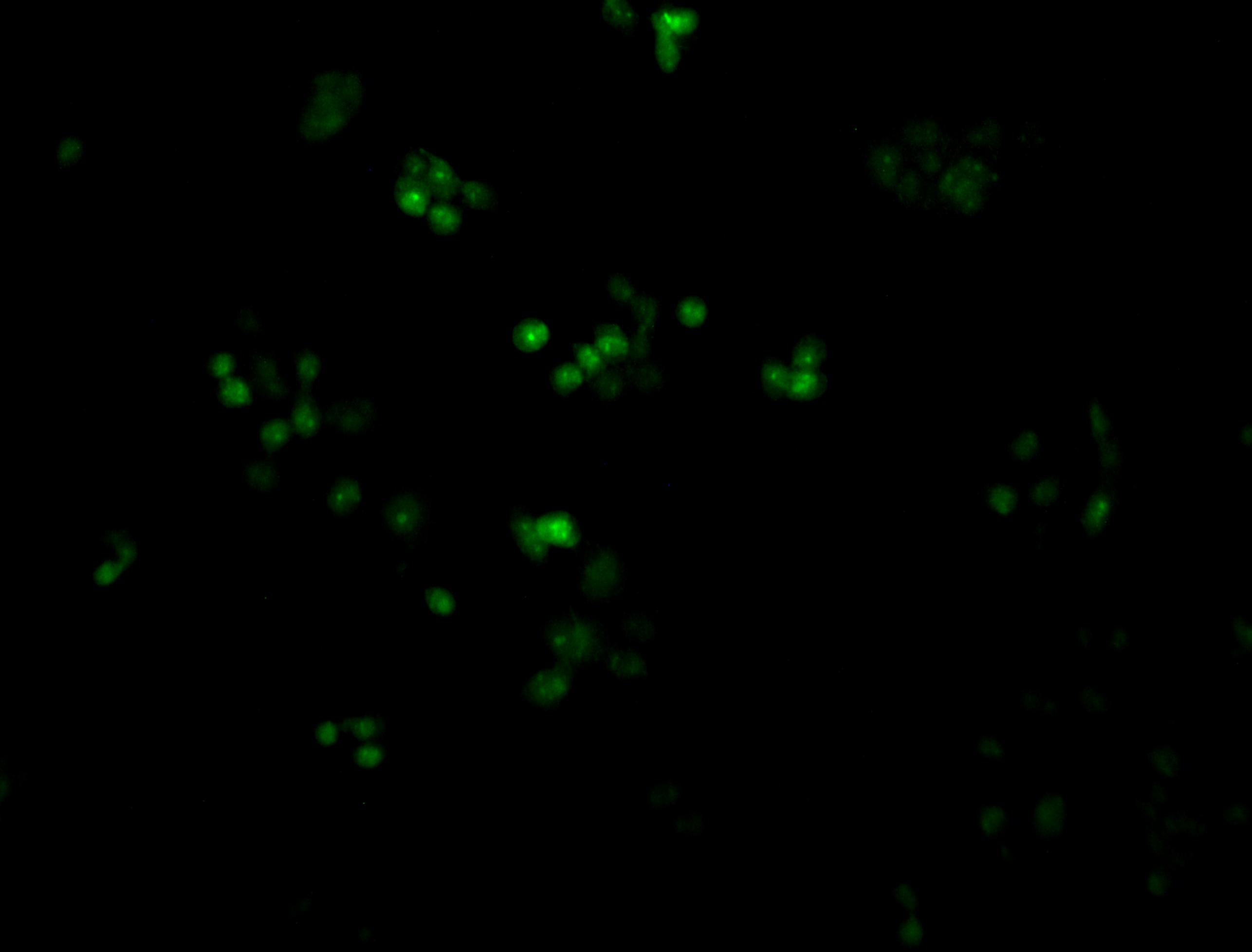

Supplement: Supplementary file 1 [file Presentation1.zip › original images-1/hr+cur+asi (8).jpg]

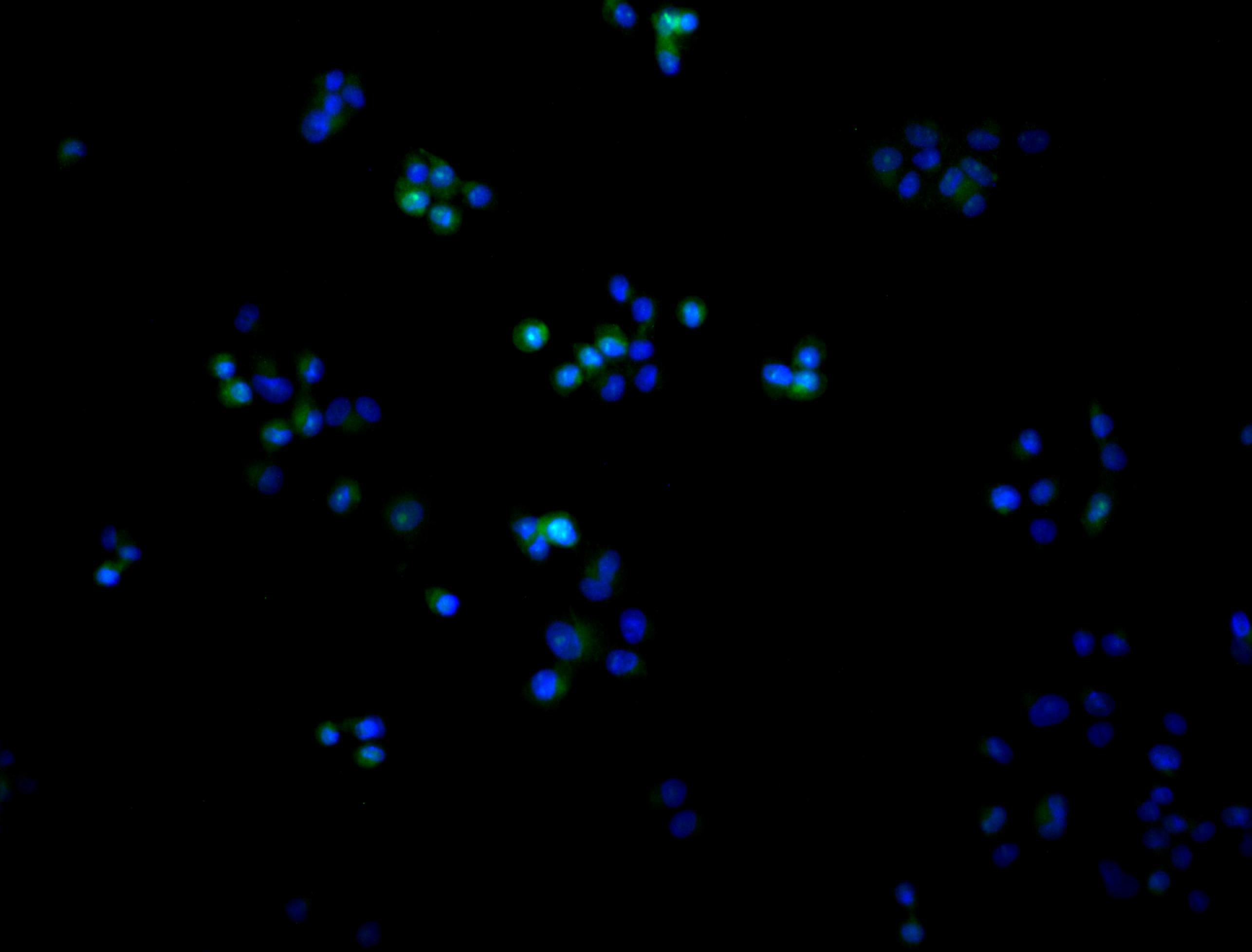

Supplement: Supplementary file 1 [file Presentation1.zip › original images-1/hr+cur+asi (9).jpg]

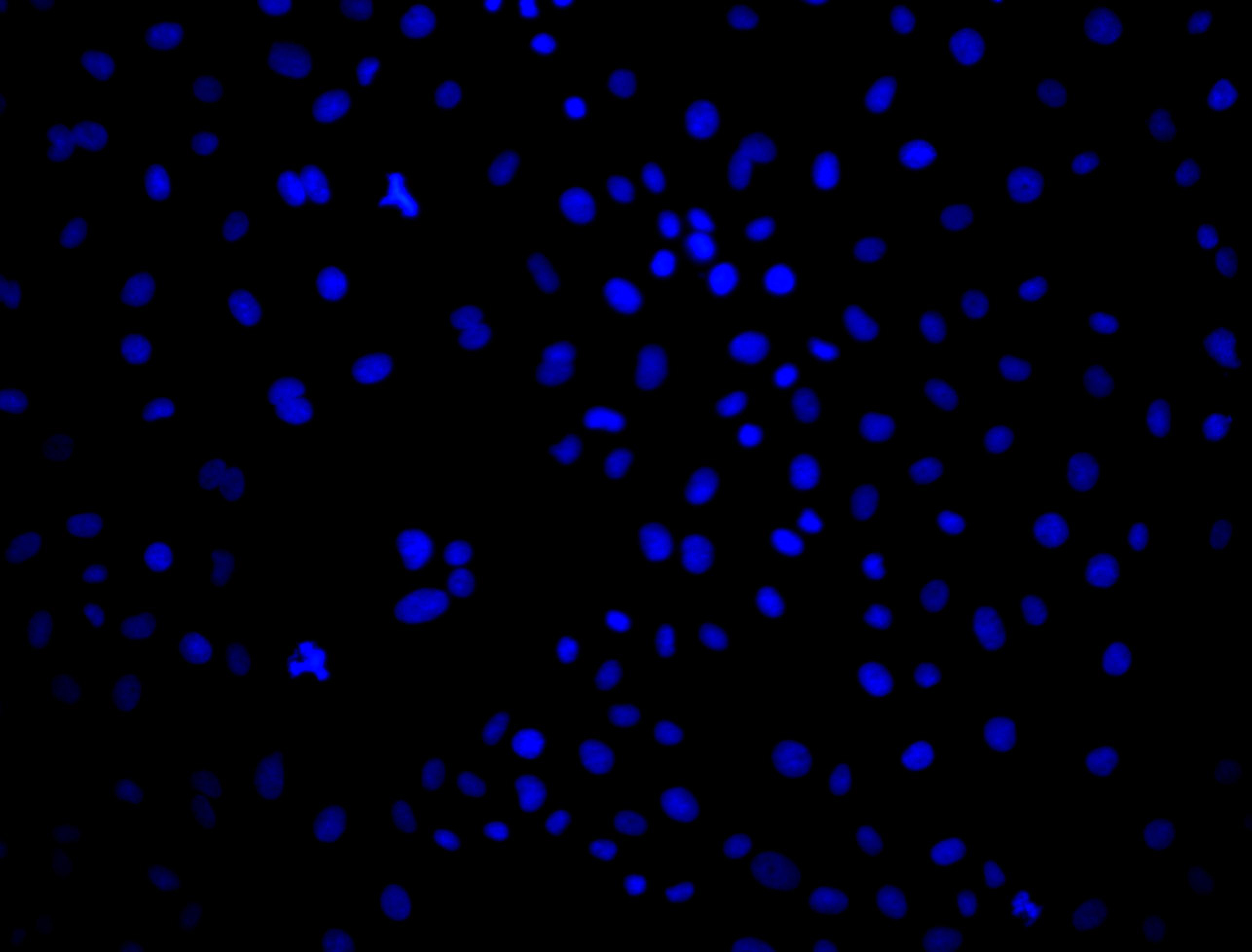

Supplement: Supplementary file 1 [file Presentation1.zip › original images-1/hr+cur+nc (7).jpg]

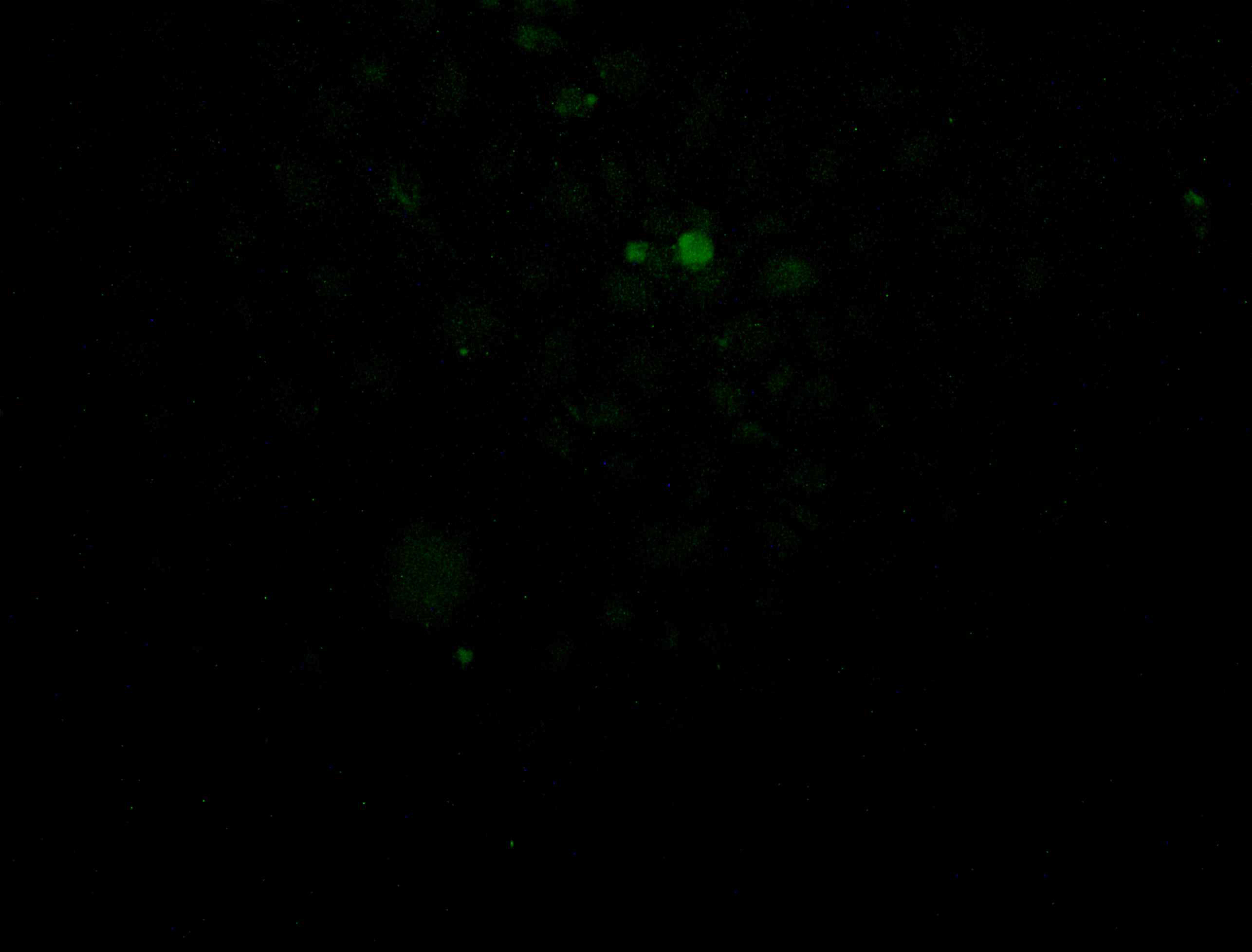

Supplement: Supplementary file 1 [file Presentation1.zip › original images-1/hr+cur+nc (8).jpg]

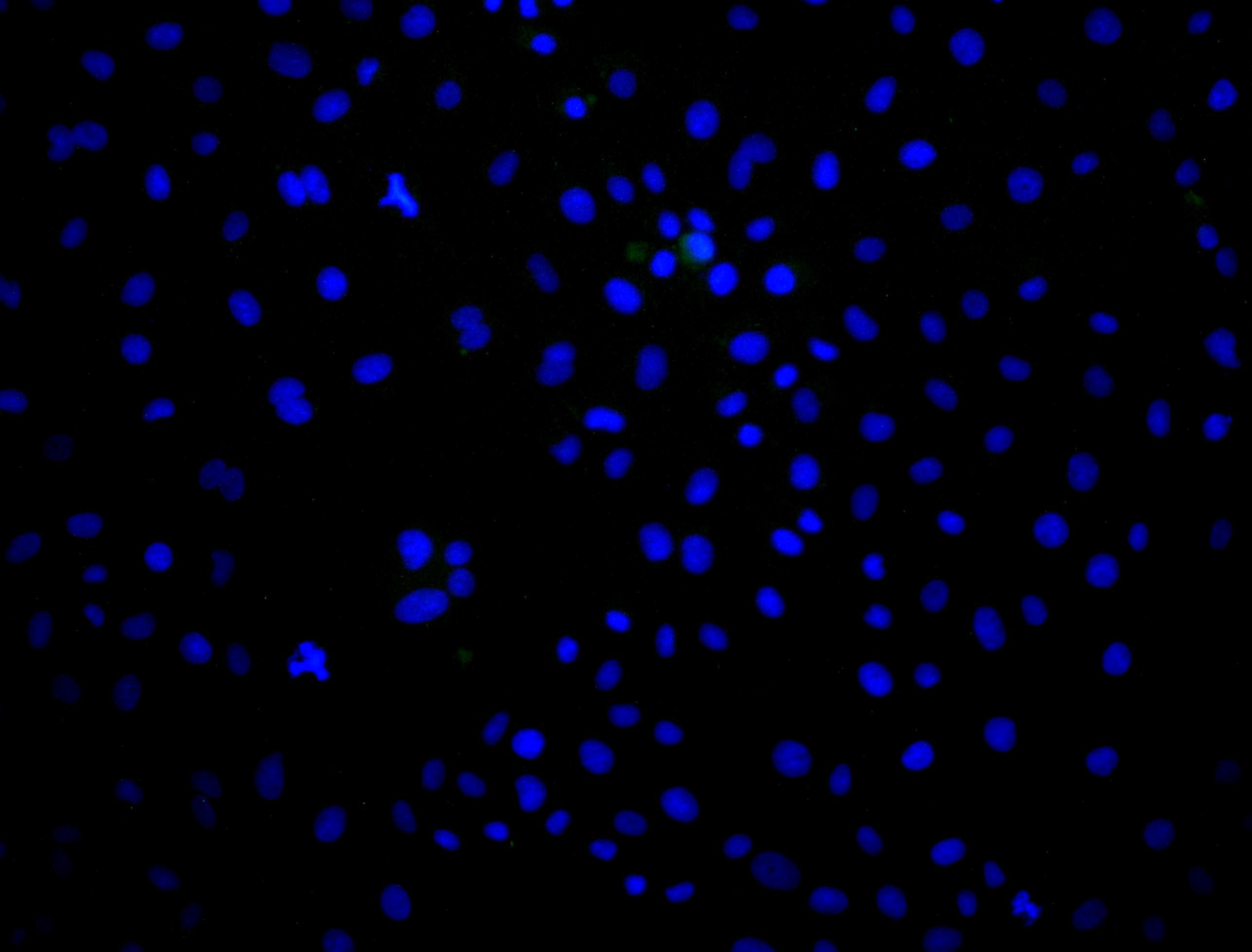

Supplement: Supplementary file 1 [file Presentation1.zip › original images-1/hr+cur+nc (9).jpg]

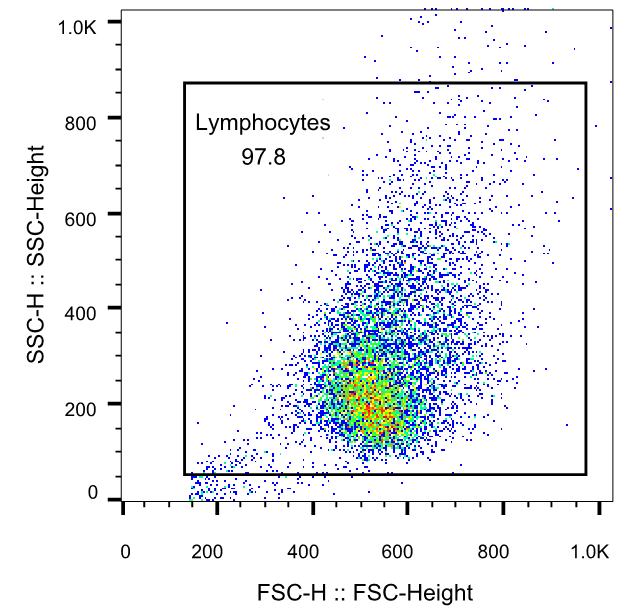

Supplement: Supplementary file 2 [file Presentation2.zip › original images-2/0.tif]

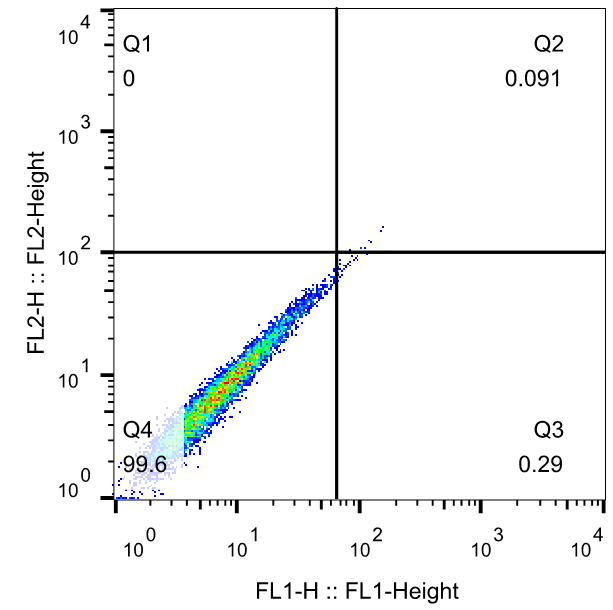

Supplement: Supplementary file 2 [file Presentation2.zip › original images-2/1.tif]

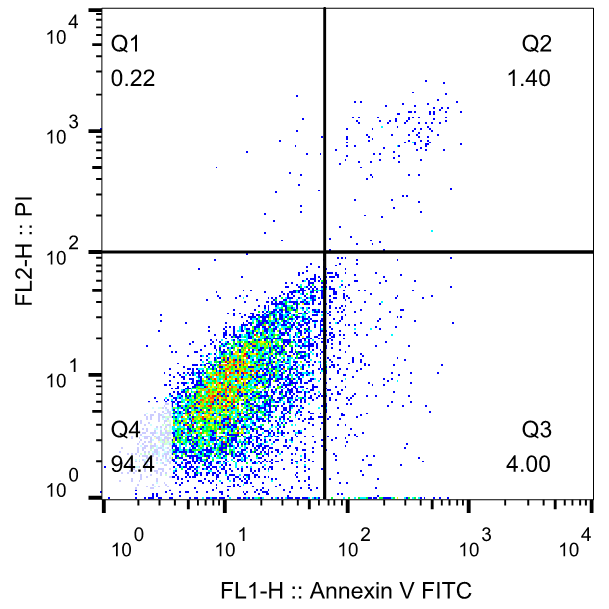

Supplement: Supplementary file 2 [file Presentation2.zip › original images-2/cell.tif]

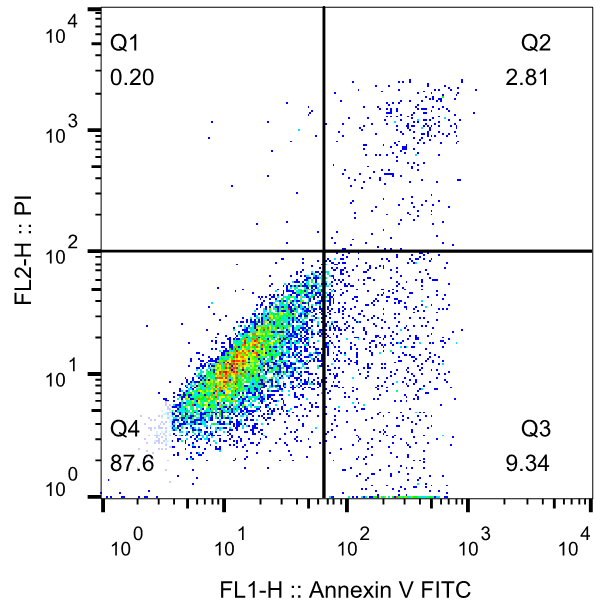

Supplement: Supplementary file 2 [file Presentation2.zip › original images-2/H+cur+Appl1siRNA+AZD5363.tif]

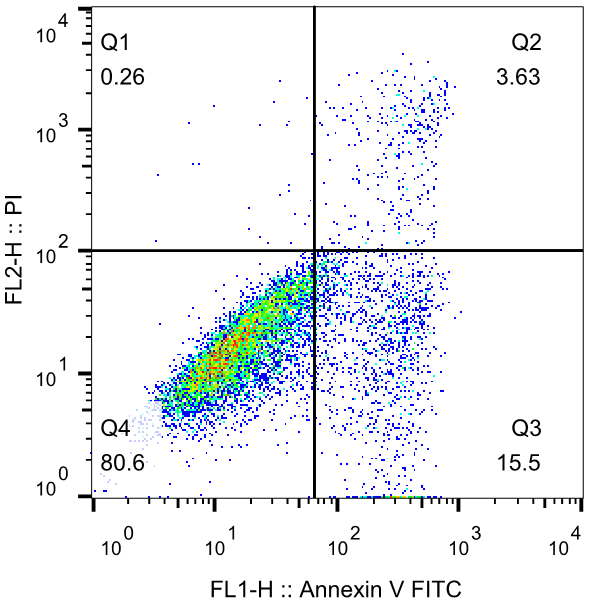

Supplement: Supplementary file 2 [file Presentation2.zip › original images-2/H+cur+Appl1siRNA.tif]

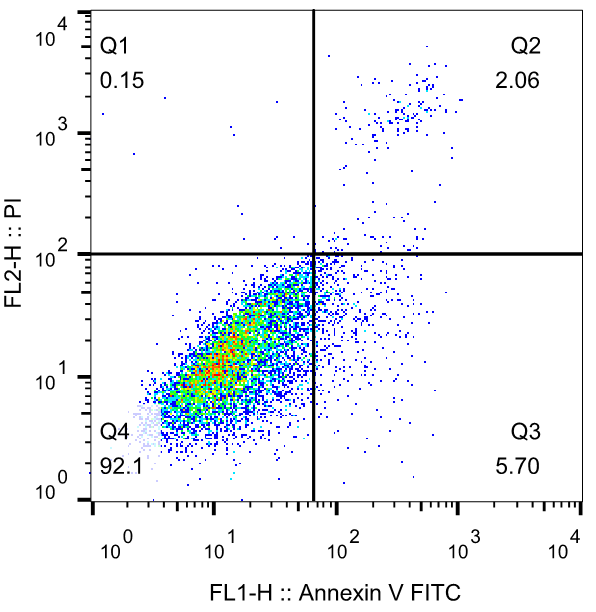

Supplement: Supplementary file 2 [file Presentation2.zip › original images-2/H+cur+NC.tif]

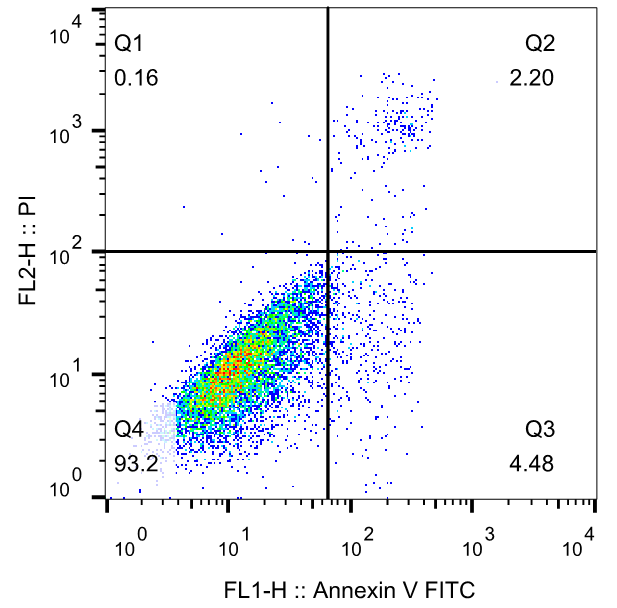

Supplement: Supplementary file 2 [file Presentation2.zip › original images-2/H+cur.tif]

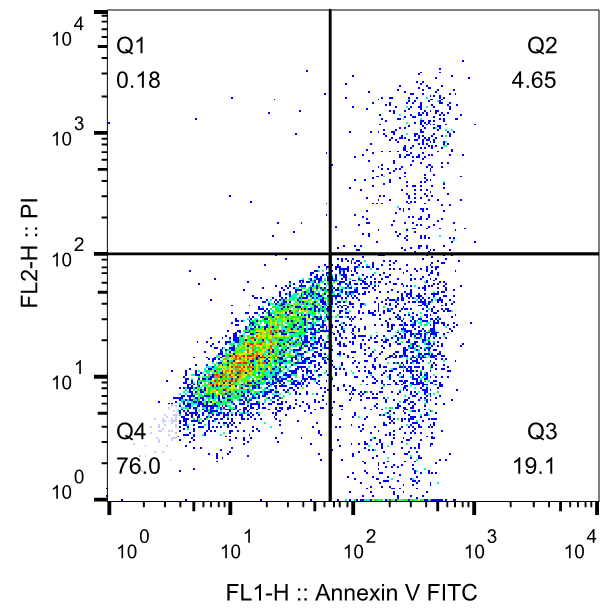

Supplement: Supplementary file 2 [file Presentation2.zip › original images-2/hr.tif]

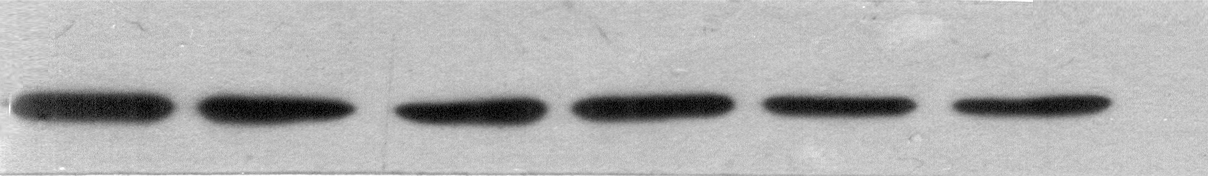

Supplement: Supplementary file 3 [file Presentation3.zip › original images-3/akt.tif]

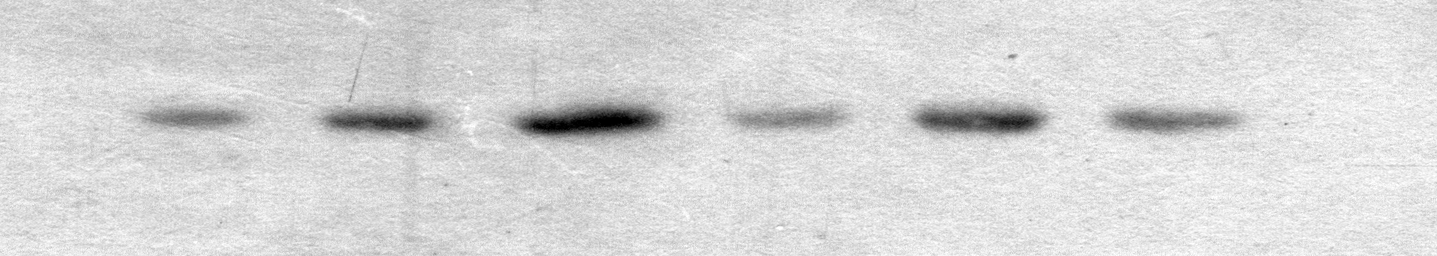

Supplement: Supplementary file 3 [file Presentation3.zip › original images-3/appl1.tif]

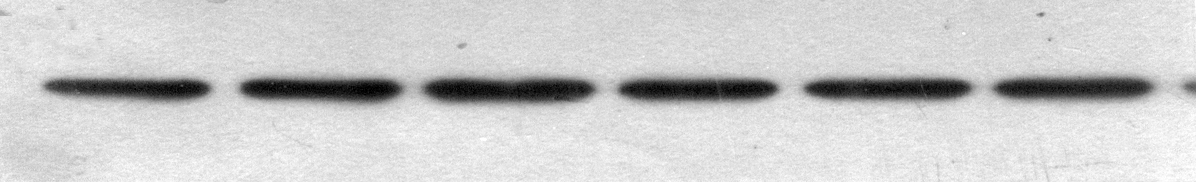

Supplement: Supplementary file 3 [file Presentation3.zip › original images-3/b3.tif]

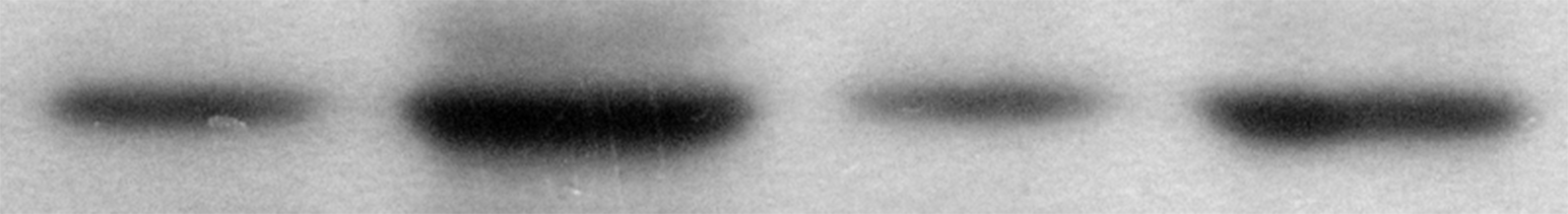

Supplement: Supplementary file 3 [file Presentation3.zip › original images-3/bax 1.tif]

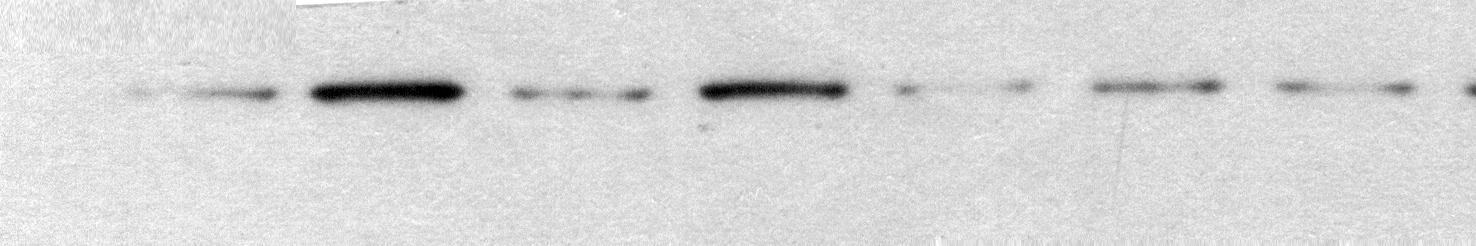

Supplement: Supplementary file 3 [file Presentation3.zip › original images-3/bax.tif]

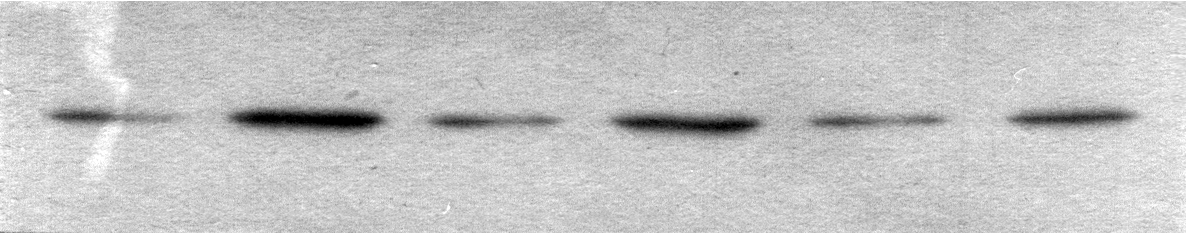

Supplement: Supplementary file 3 [file Presentation3.zip › original images-3/casp.tif]

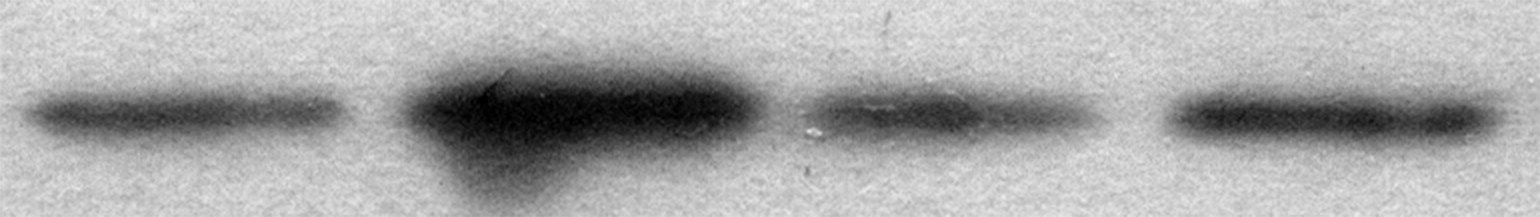

Supplement: Supplementary file 3 [file Presentation3.zip › original images-3/caspase3 1.tif]

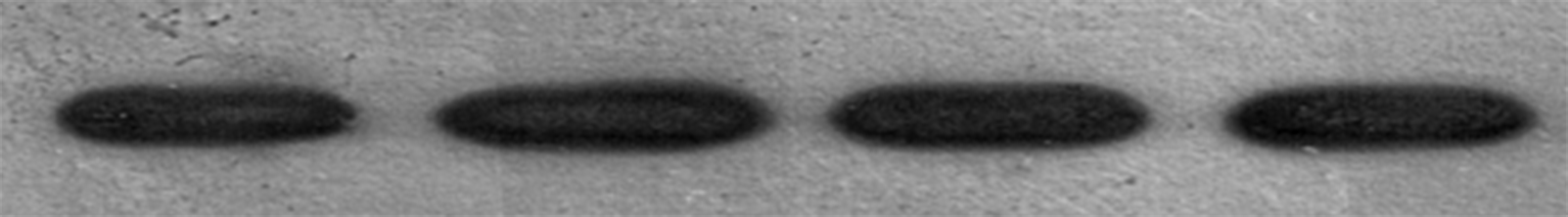

Supplement: Supplementary file 3 [file Presentation3.zip › original images-3/gadph1.tif]

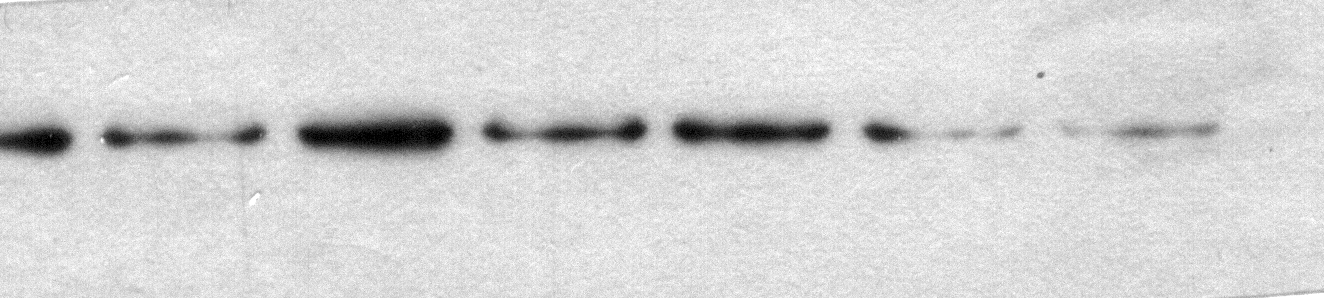

Supplement: Supplementary file 3 [file Presentation3.zip › original images-3/p-akt.tif]

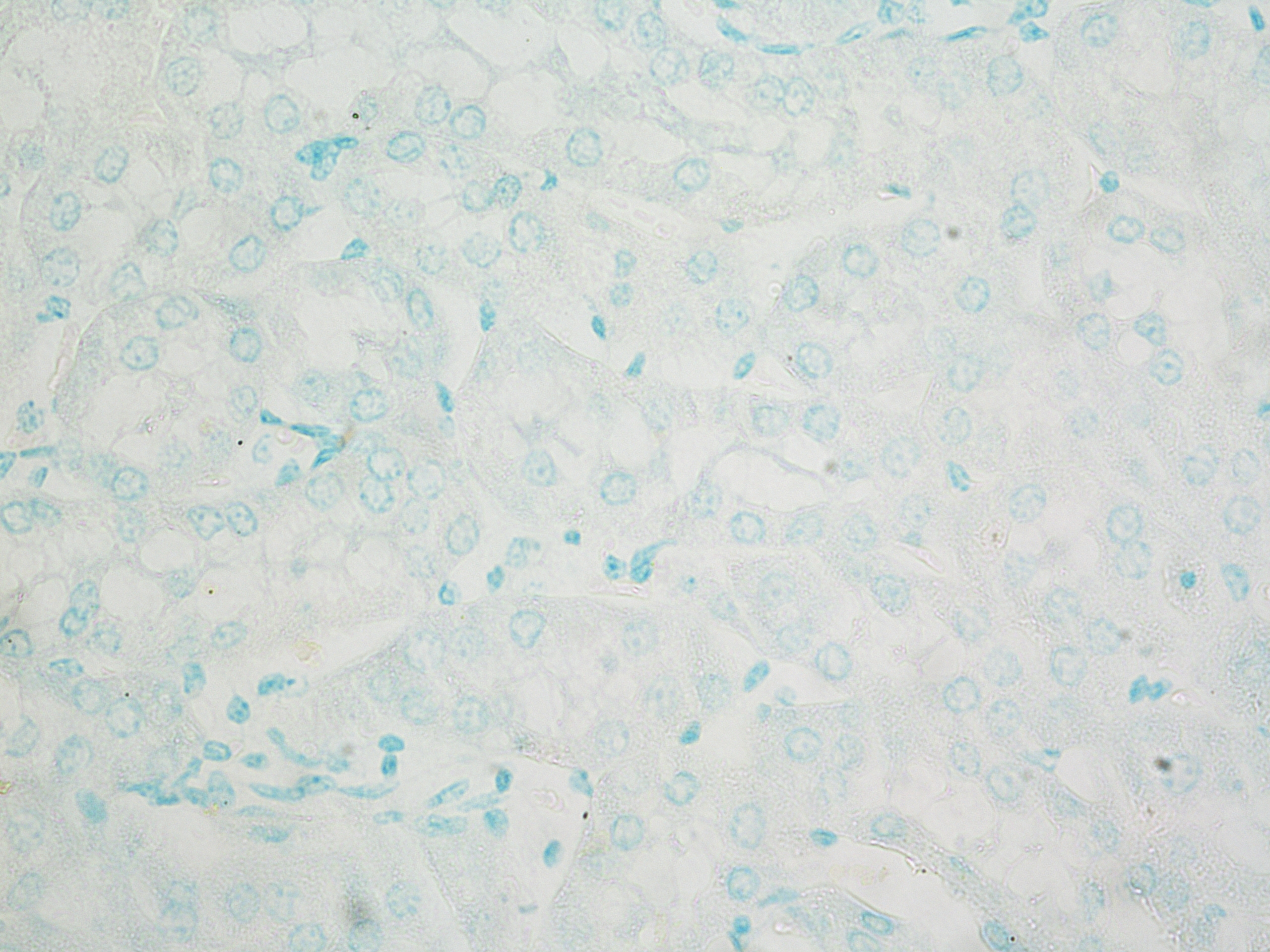

Supplement: Supplementary file 4 [file Presentation4.zip › original images-4/curcumin1 (2).jpg]

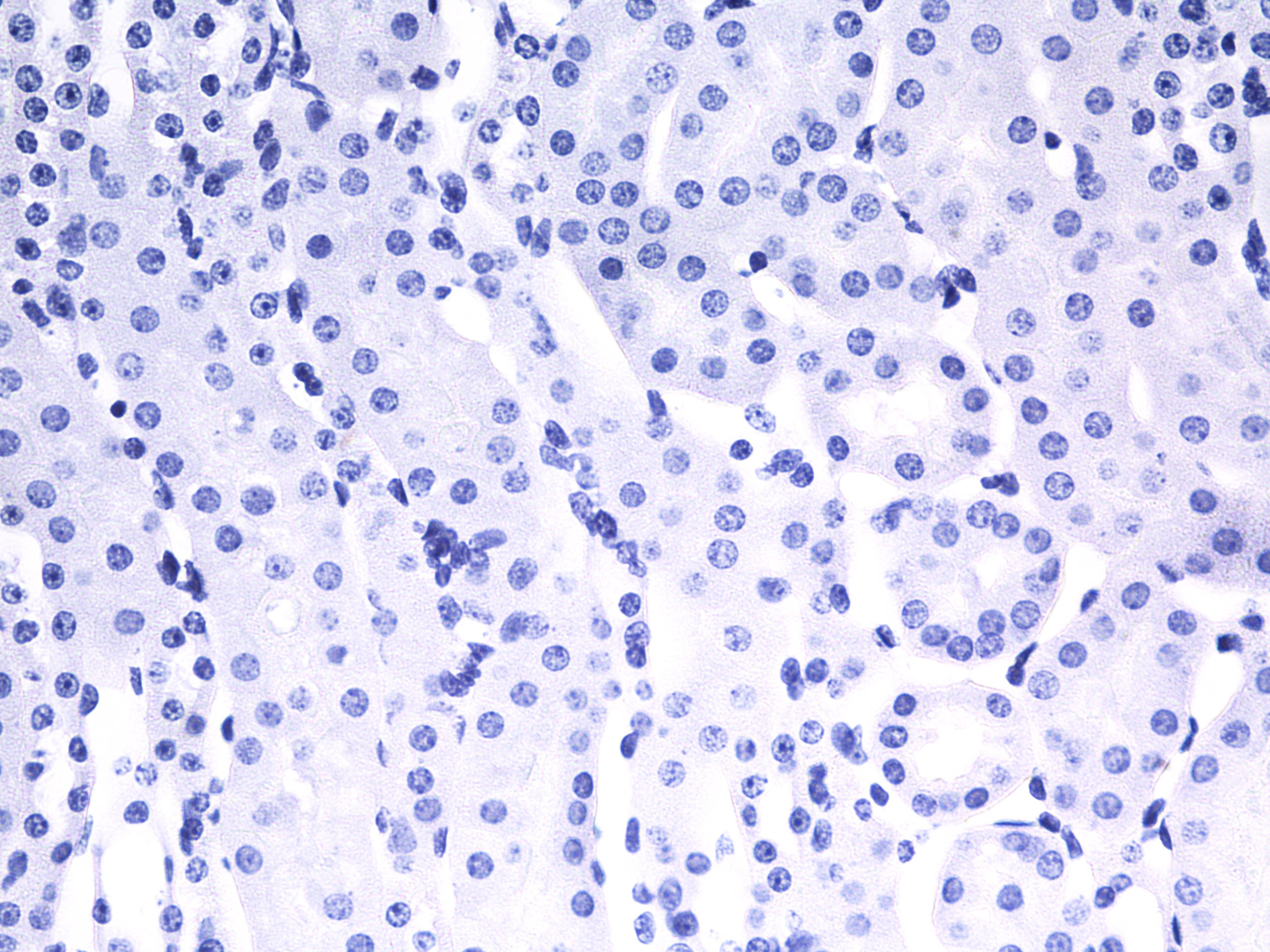

Supplement: Supplementary file 4 [file Presentation4.zip › original images-4/Curcumin1 (3).jpg]

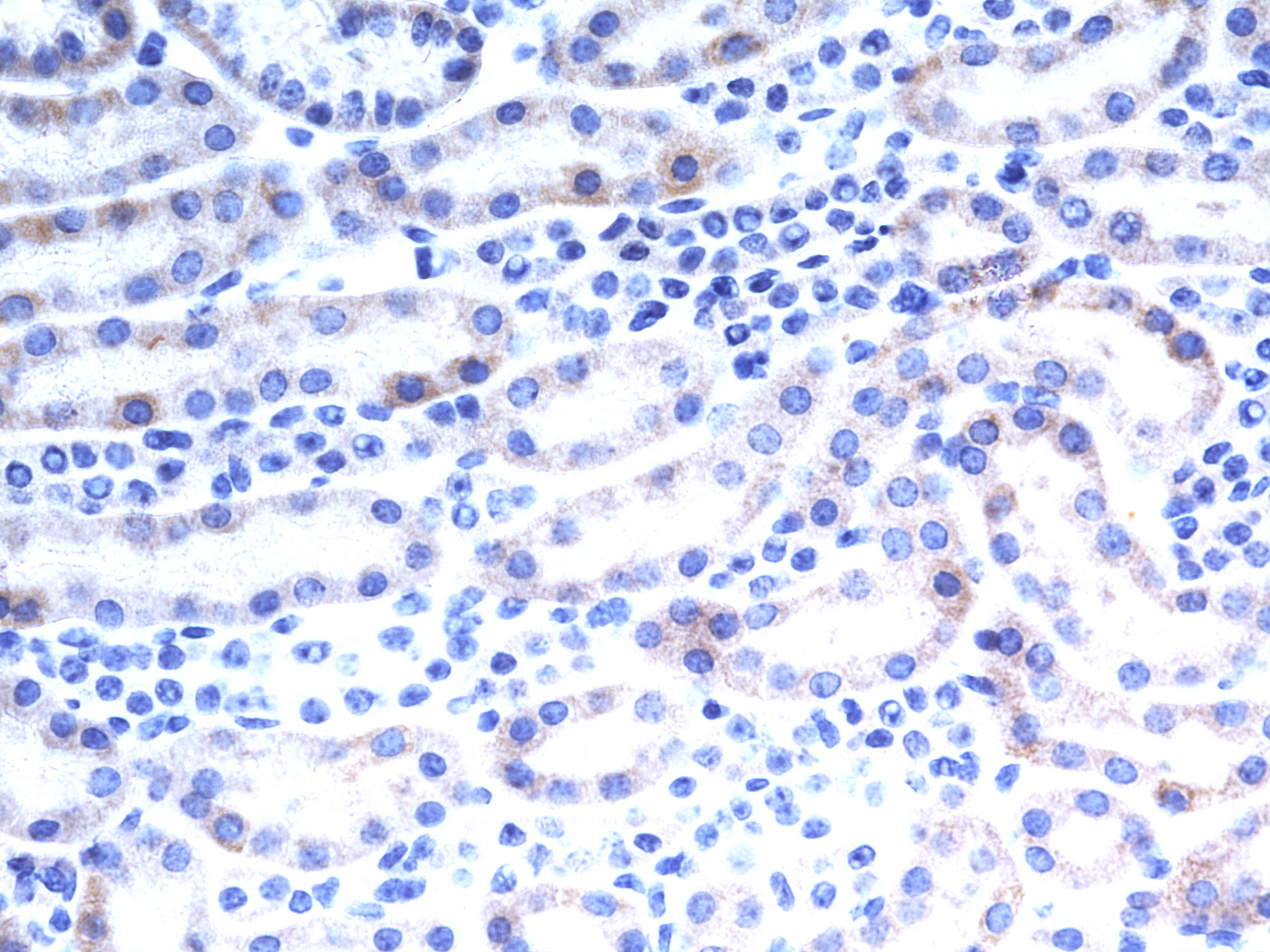

Supplement: Supplementary file 4 [file Presentation4.zip › original images-4/curcumin1 .jpg]

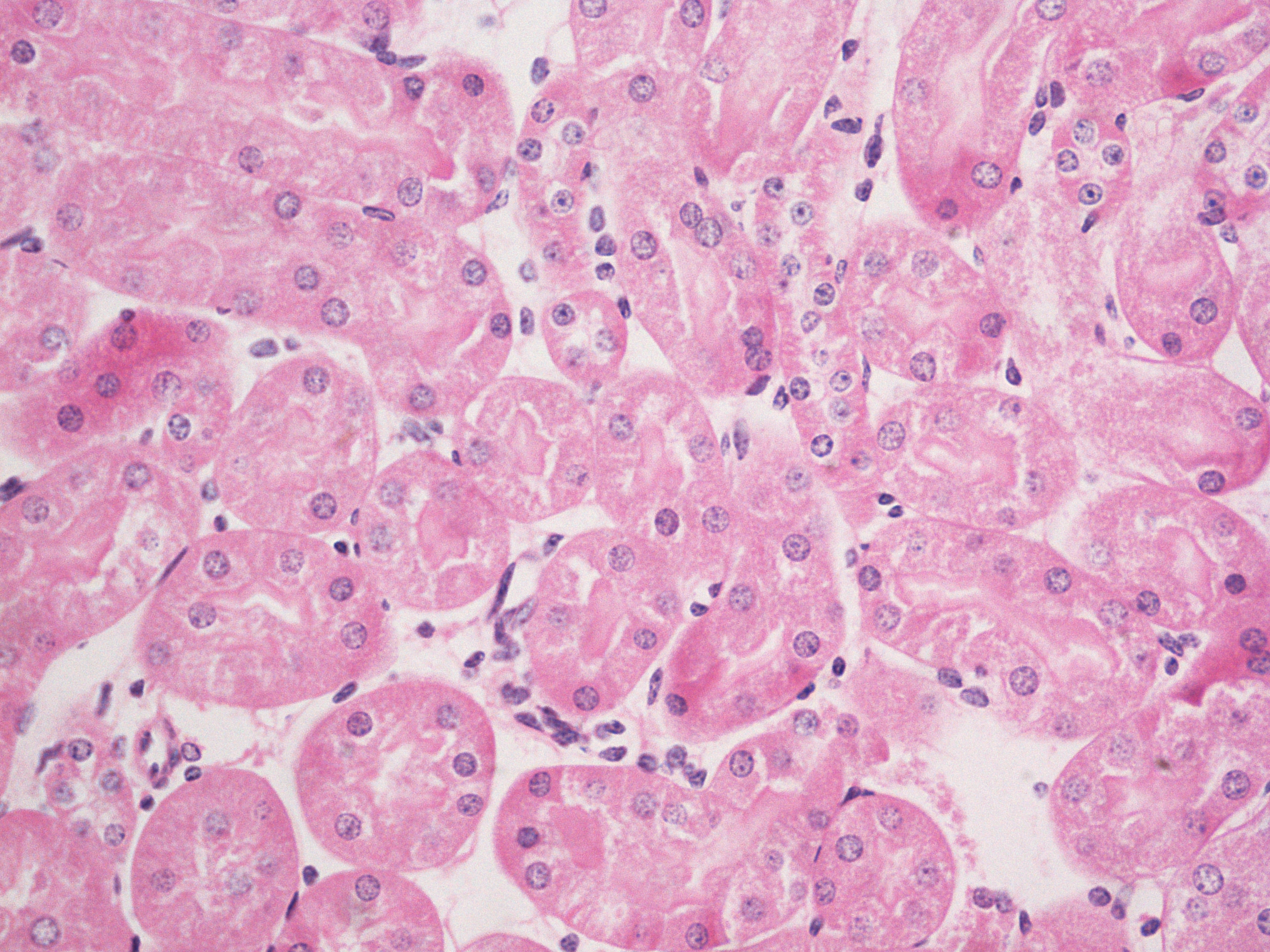

Supplement: Supplementary file 4 [file Presentation4.zip › original images-4/curcumin1.jpg]

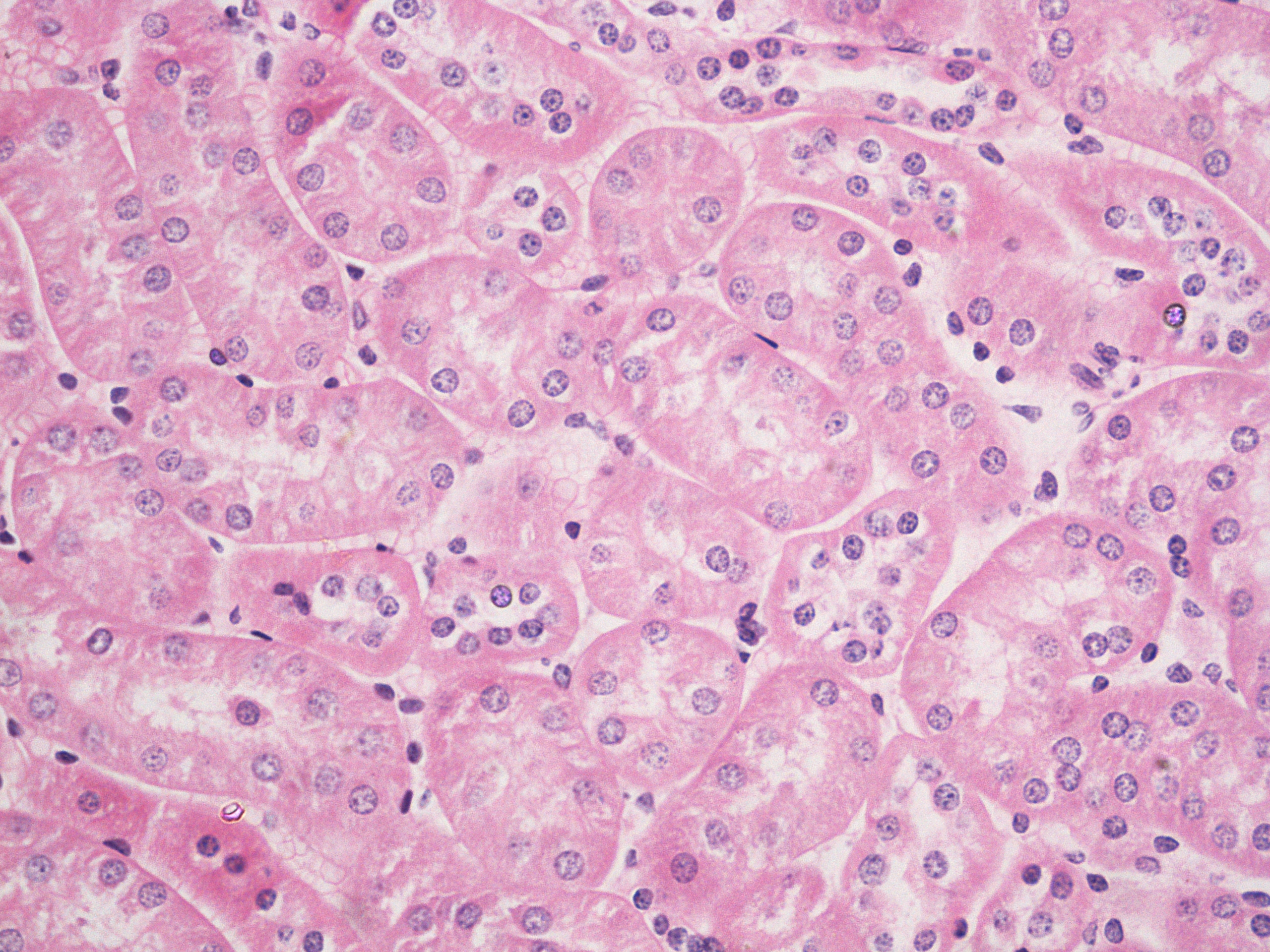

Supplement: Supplementary file 5 [file Presentation5.zip › original images-5/con1 (2).jpg]

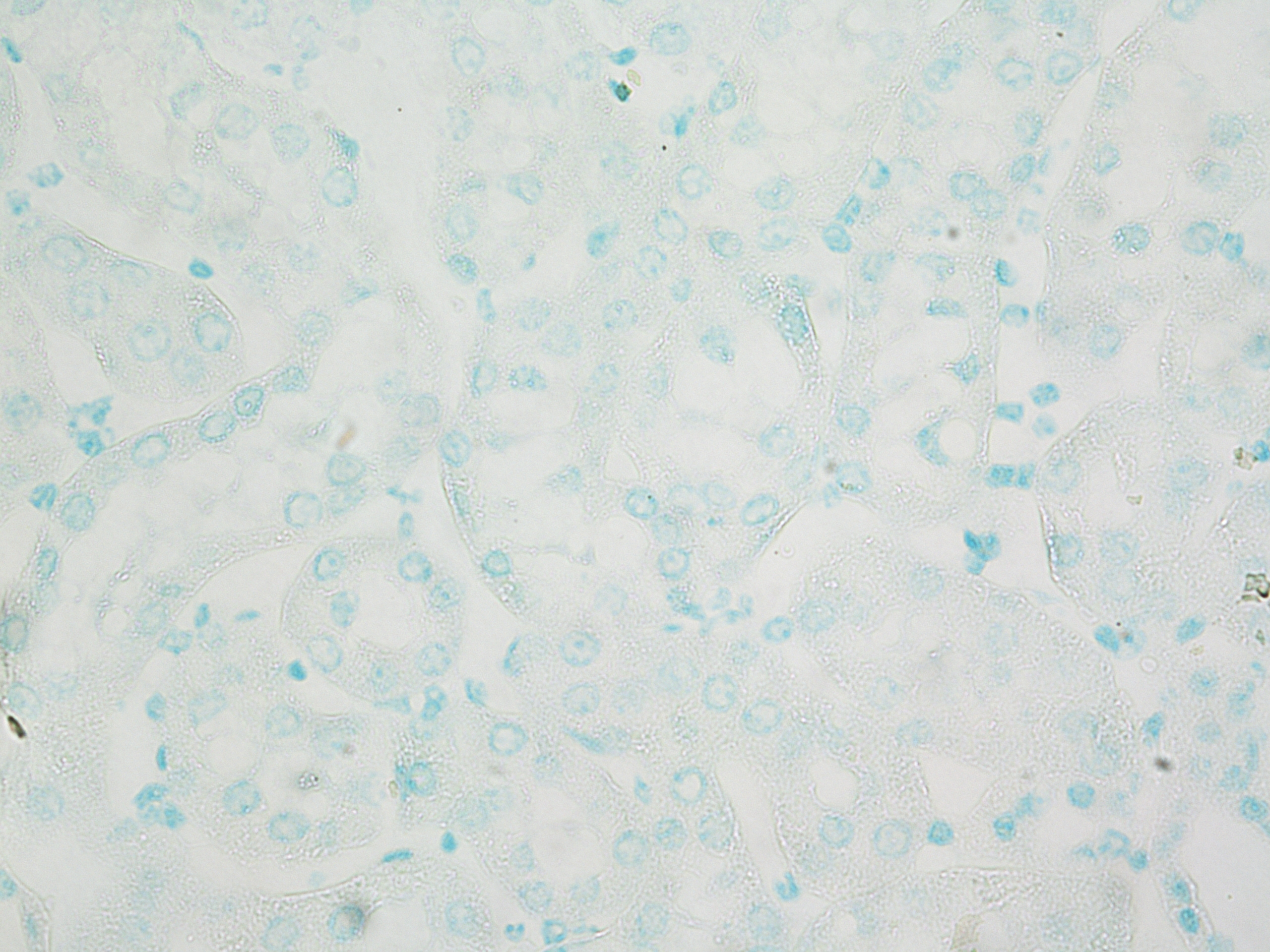

Supplement: Supplementary file 5 [file Presentation5.zip › original images-5/con1 (3).jpg]

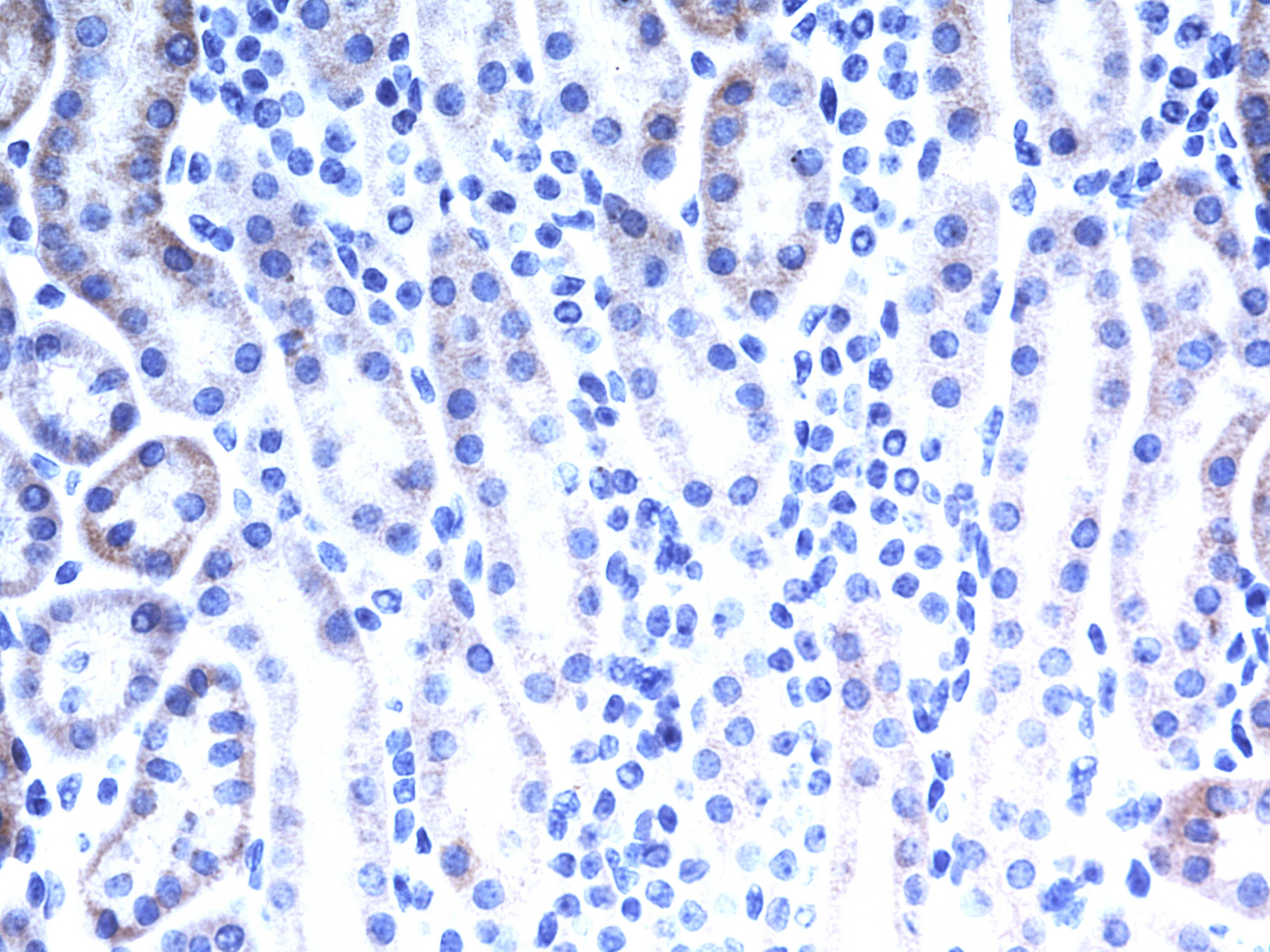

Supplement: Supplementary file 5 [file Presentation5.zip › original images-5/con1 .jpg]

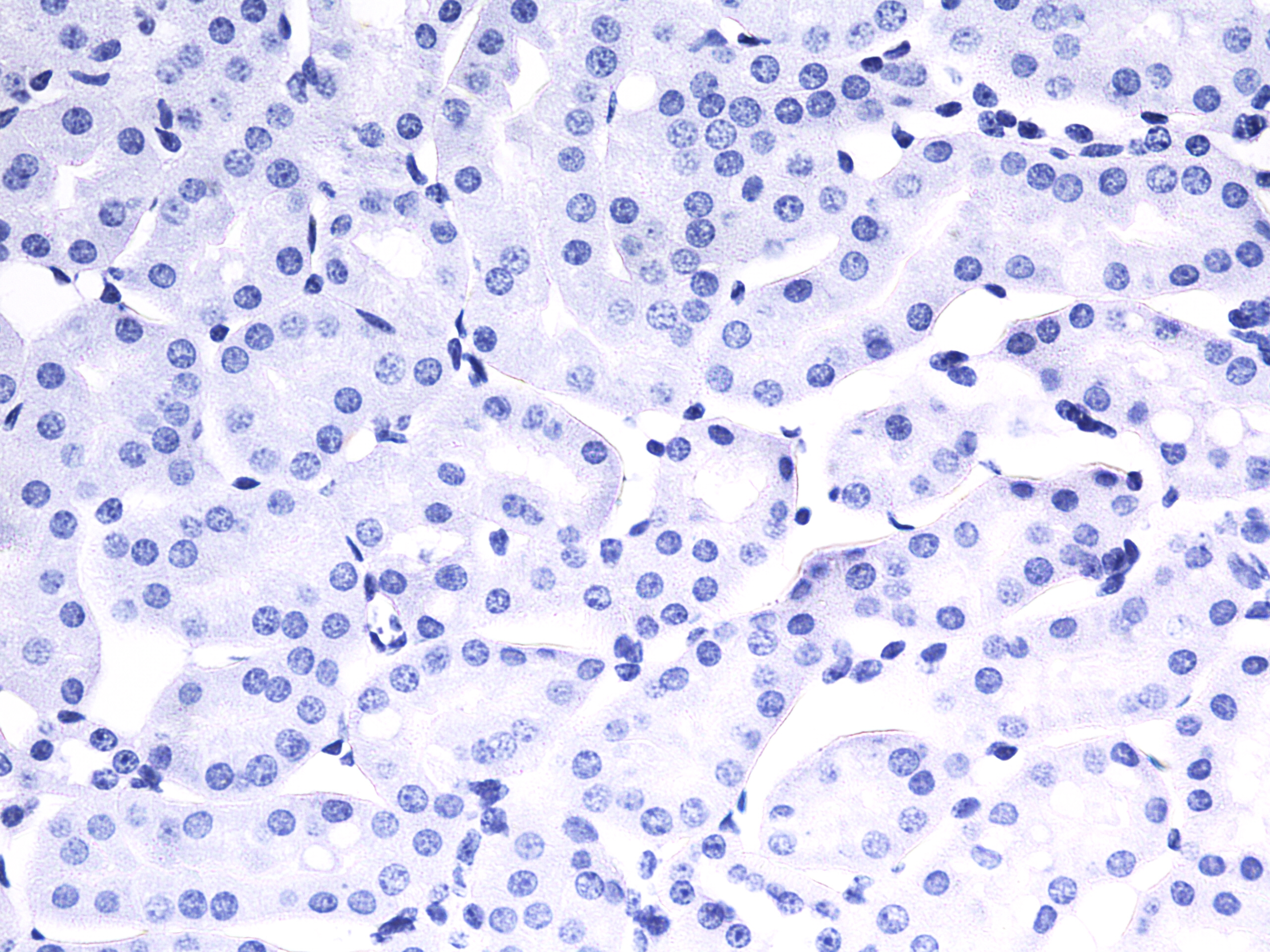

Supplement: Supplementary file 5 [file Presentation5.zip › original images-5/Con1.jpg]

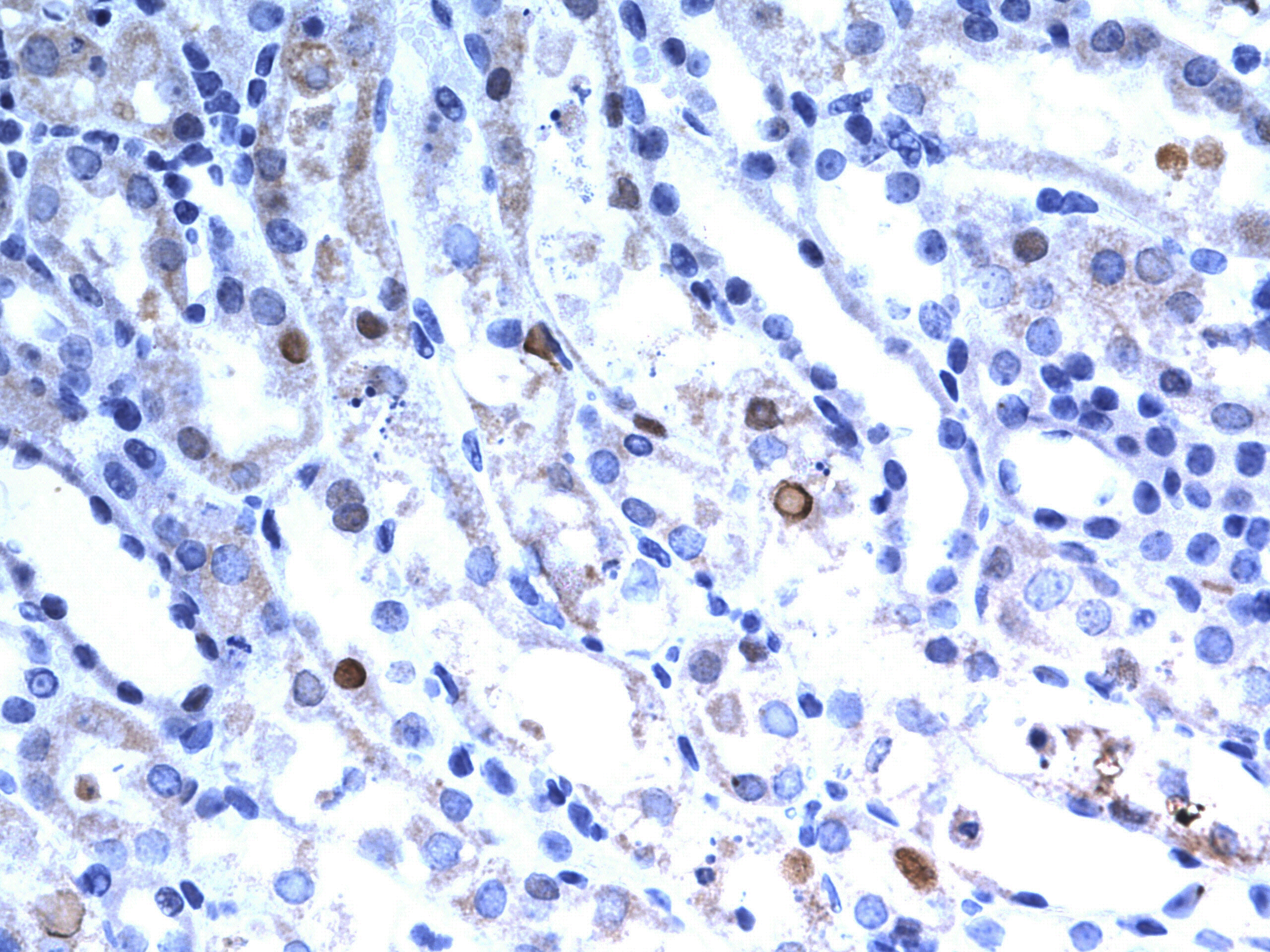

Supplement: Supplementary file 6 [file Presentation6.zip › original images-6/IR+Curcumin 1.jpg]

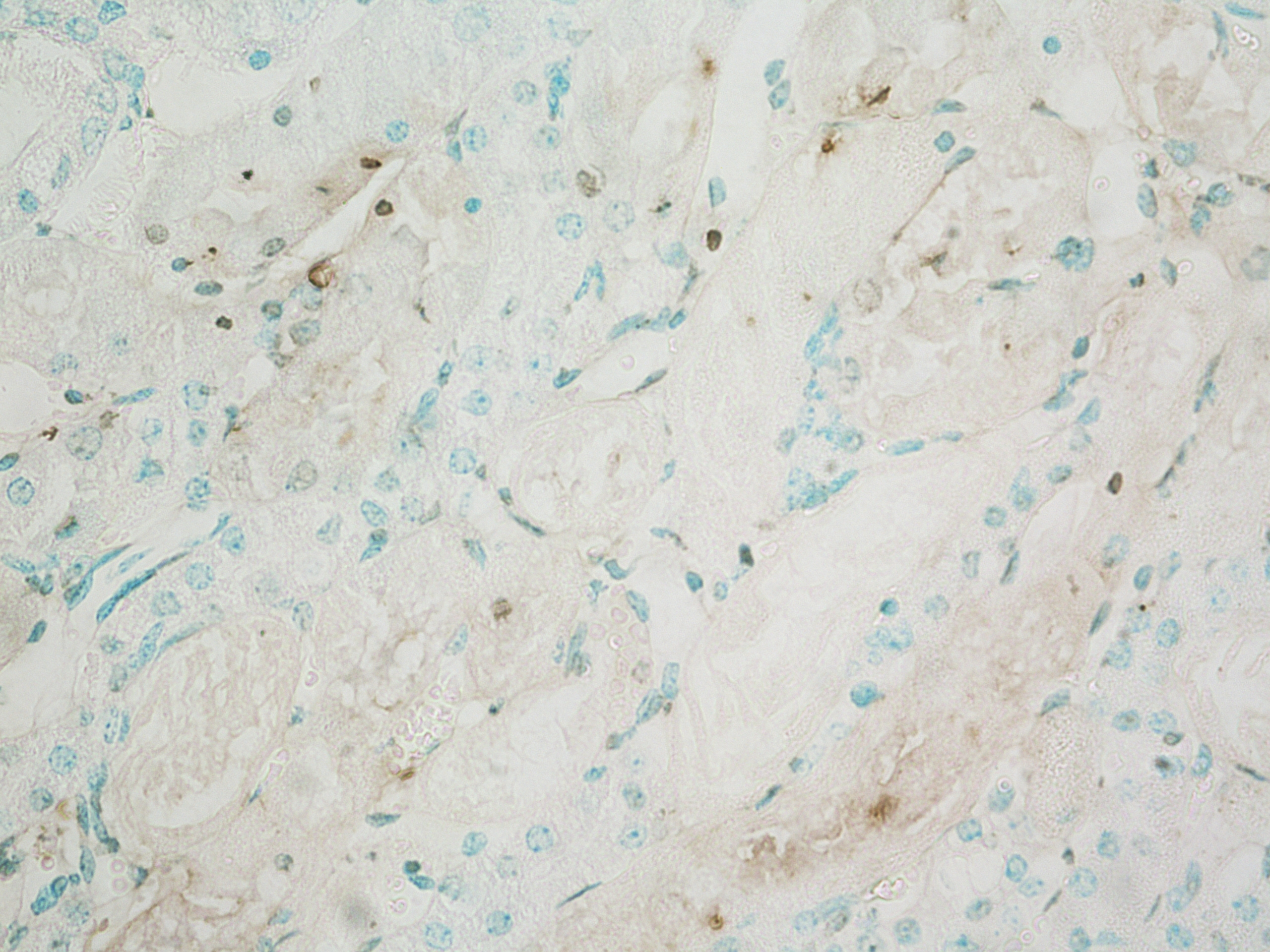

Supplement: Supplementary file 6 [file Presentation6.zip › original images-6/IR+curcumin1 (2).jpg]

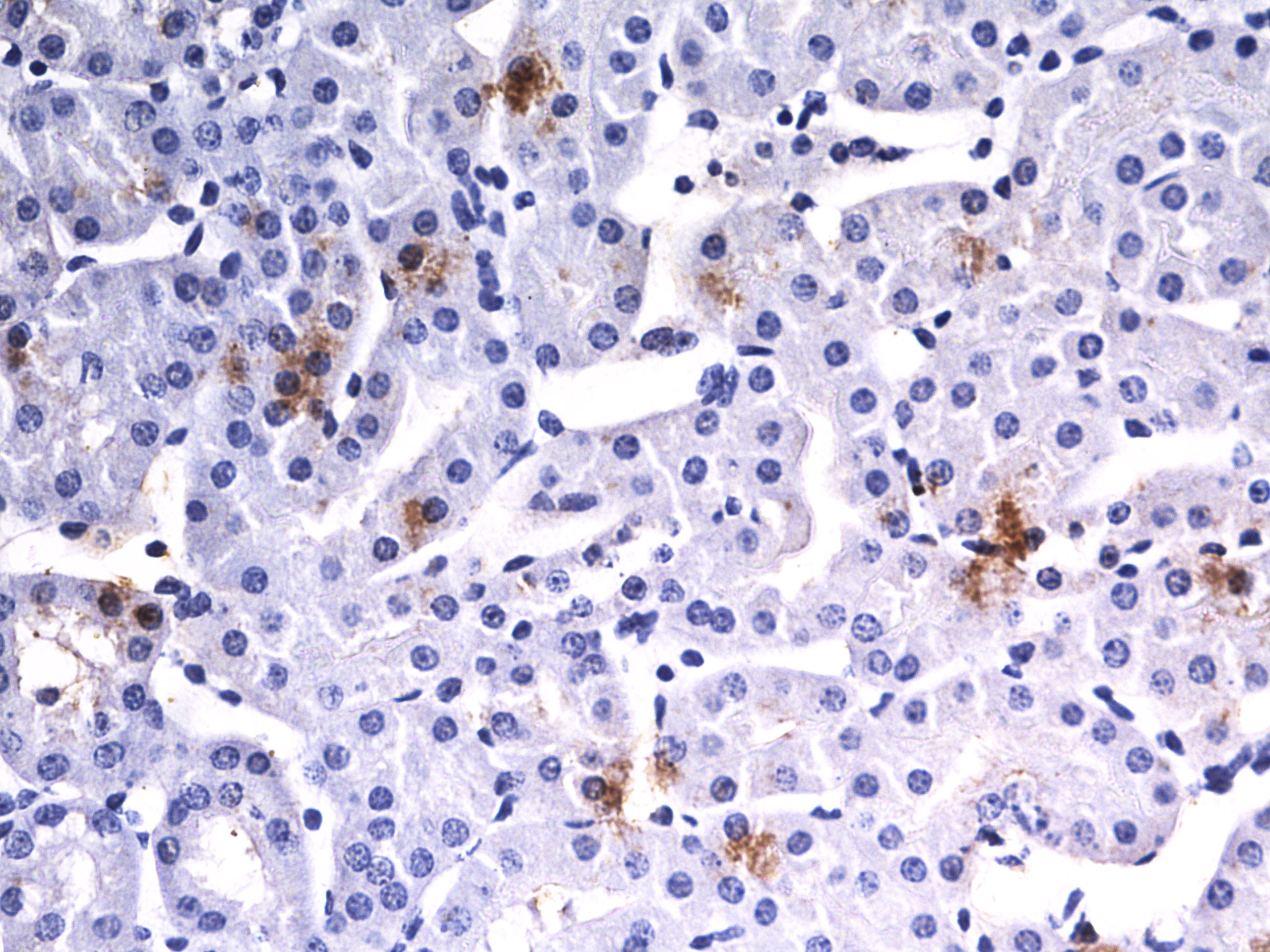

Supplement: Supplementary file 6 [file Presentation6.zip › original images-6/IR+curcumin1 (3).jpg]

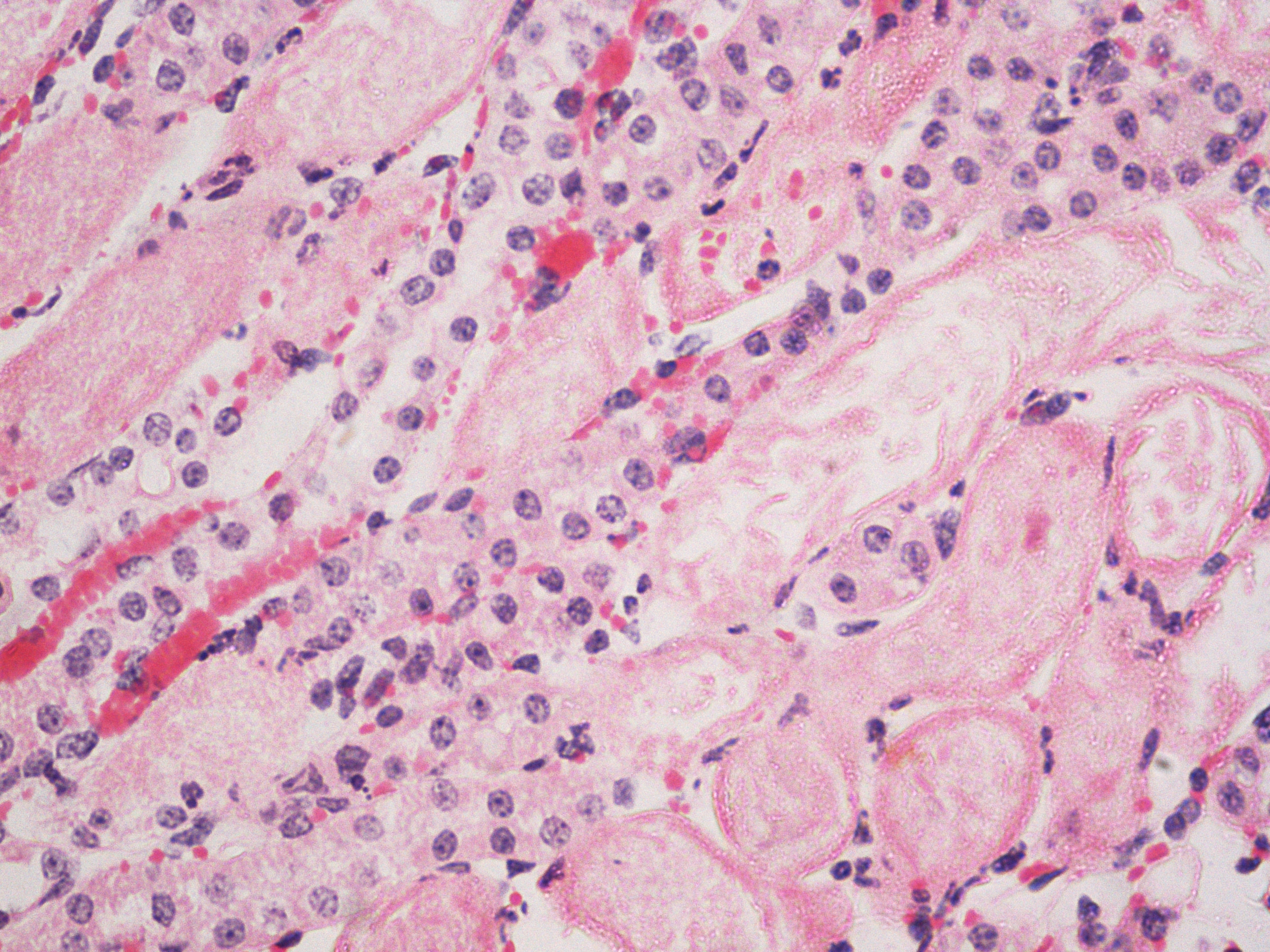

Supplement: Supplementary file 6 [file Presentation6.zip › original images-6/IR+curcumin1.jpg]

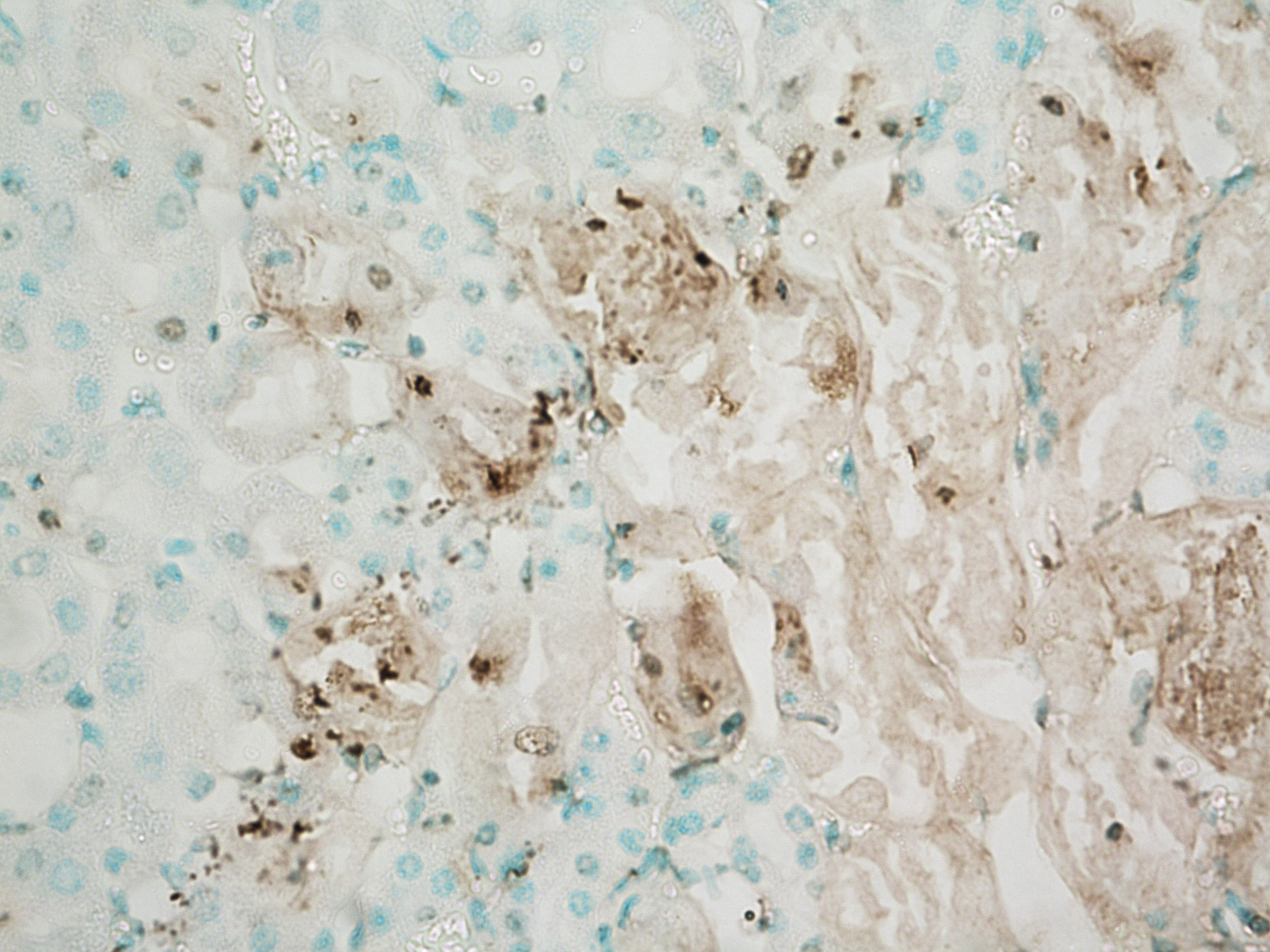

Supplement: Supplementary file 7 [file Presentation7.zip › original images-7/IR1 (2).jpg]

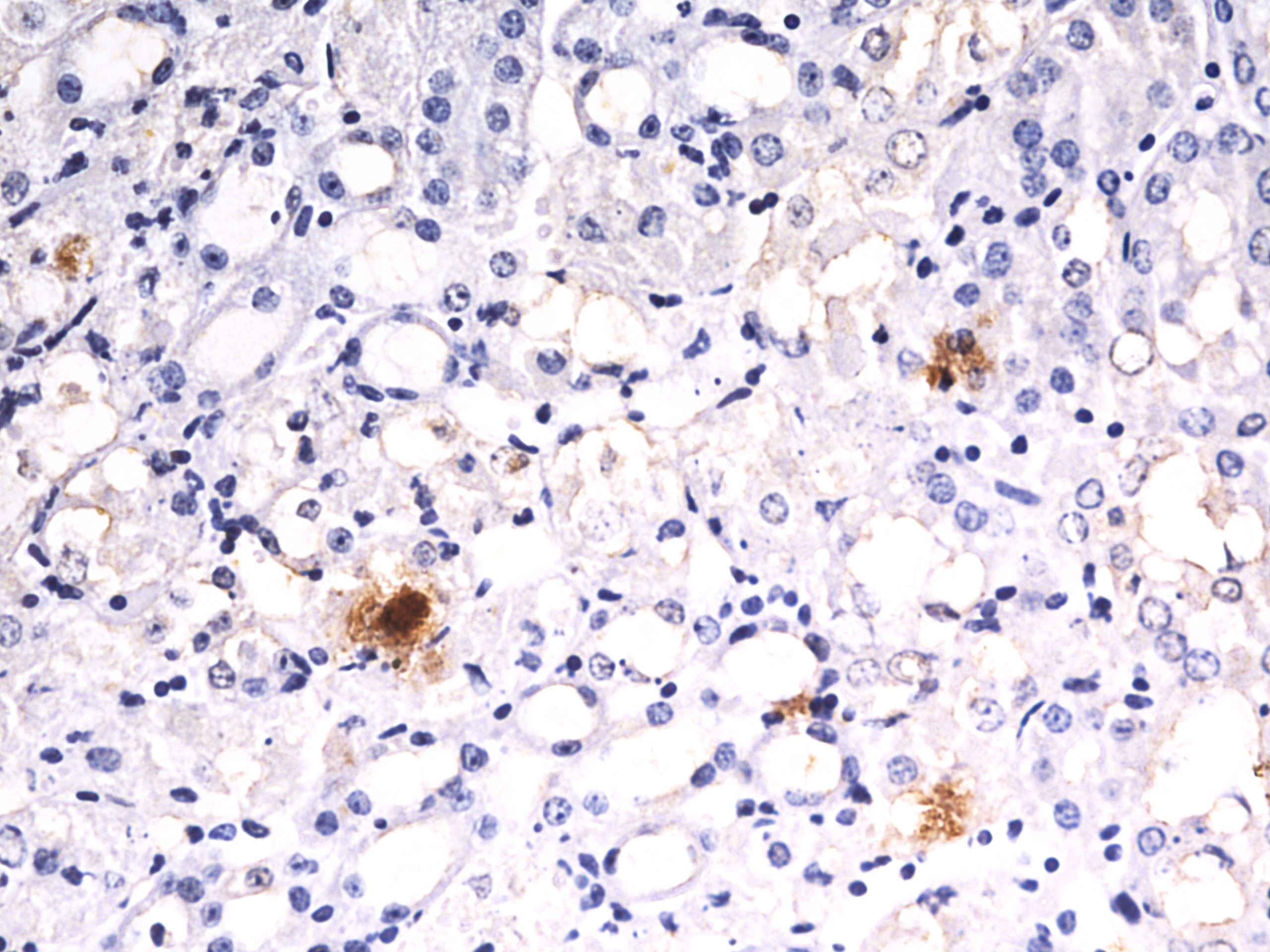

Supplement: Supplementary file 7 [file Presentation7.zip › original images-7/IR1 (3).jpg]

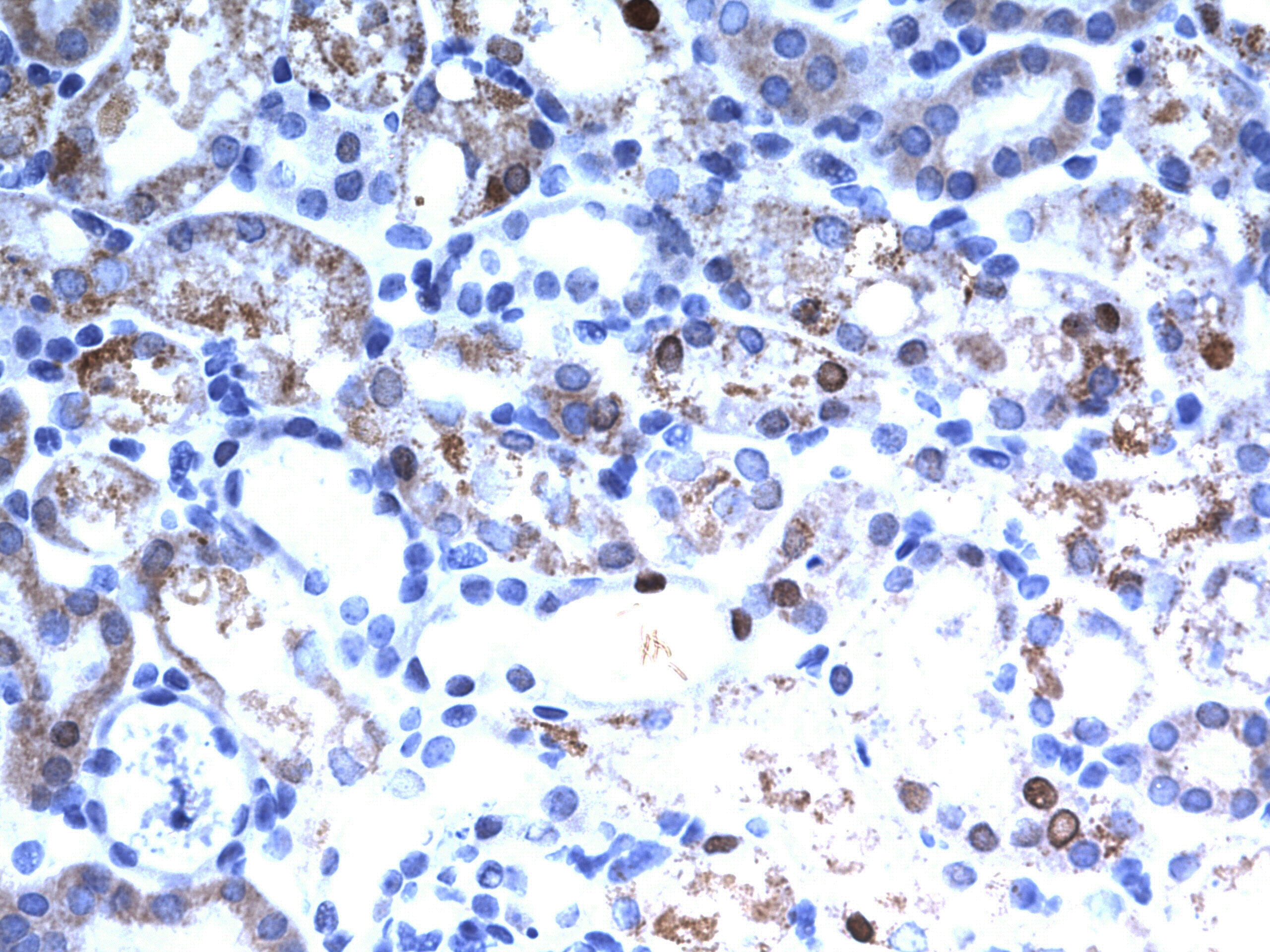

Supplement: Supplementary file 7 [file Presentation7.zip › original images-7/IR1.jpg]

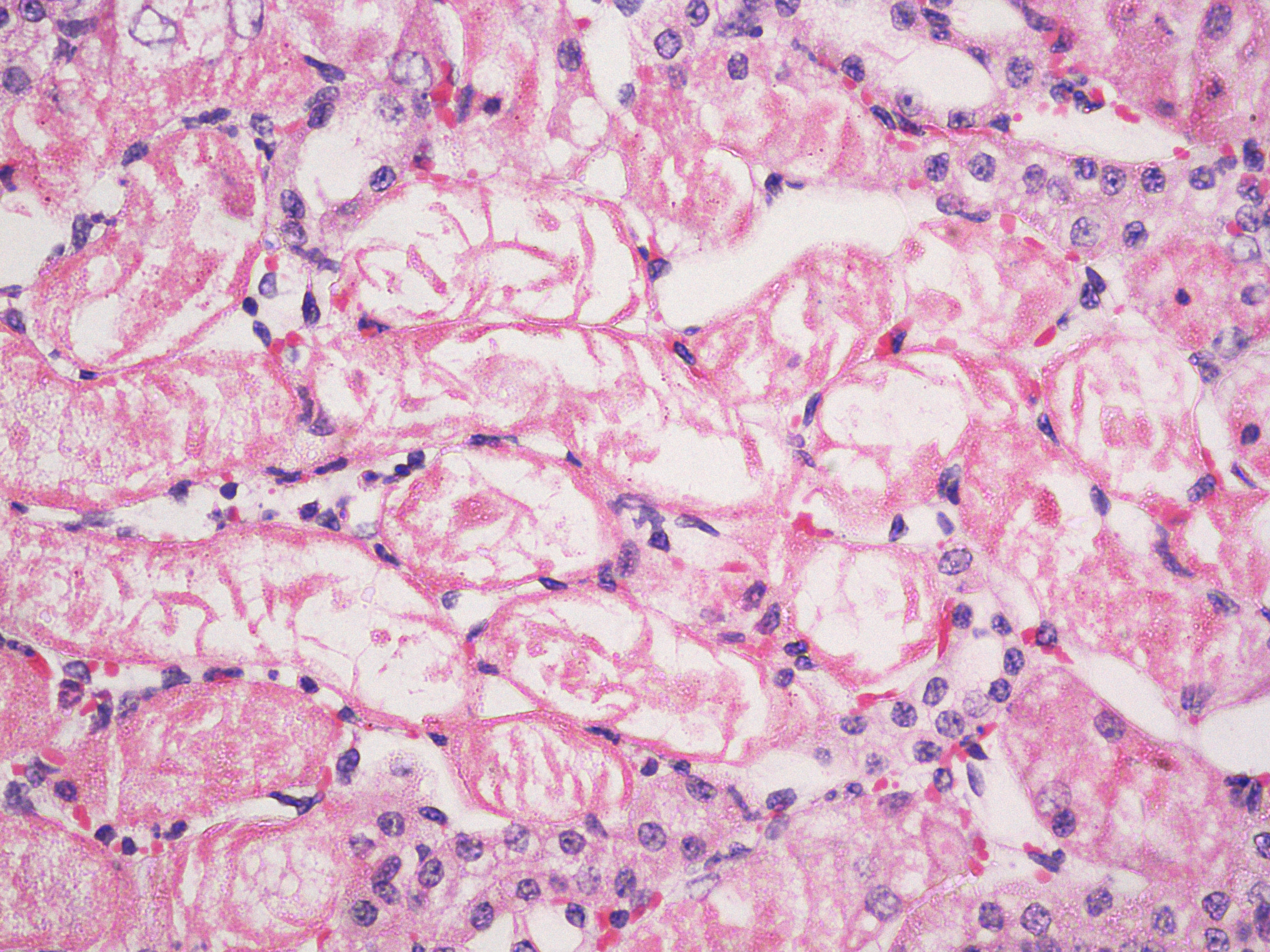

Supplement: Supplementary file 7 [file Presentation7.zip › original images-7/IRI-1.jpg]
